# Combustion Behavior of Cellulose Ester Fibrous Bundles from Used Cigarette Filters: Kinetic Analysis Study

**DOI:** 10.3390/polym16111480

**Published:** 2024-05-23

**Authors:** Filip Veljković, Vladimir Dodevski, Milena Marinović-Cincović, Suzana Veličković, Bojan Janković

**Affiliations:** Institute of Nuclear Sciences—National Institute of the Republic of Serbia, University of Belgrade, “Vinča”, Mike Petrovića Alasa 12-14, P.O. Box 522, 11001 Belgrade, Serbia; filipveljkovic@vinca.rs (F.V.); milena@vinca.rs (M.M.-C.); vsuzana@vinca.rs (S.V.)

**Keywords:** cellulose diacetate (CDA), kinetic analysis, combustion performance, secondary crystallization, non-banded spherulites, autocatalytic mechanism, value-added chemicals, isothermal prediction

## Abstract

This study is focused on the detailed examination of the combustion properties and kinetic analysis of a cellulose acetate fibrous bundle (CAFB), separated from used cigarette filters. It was shown that the faster rate of CAFB heating allows a large amount of heat to be supplied to a combustion system in the initial stages, where the increase in heating rate has a positive response to ignition behavior. The best combustion stability of CAFB is achieved at the lowest heating rate. Through the use of different kinetic methods, it was shown that combustion takes place through two series of consecutive reaction steps and one independent single-step reaction. By optimizing the kinetic parameters within the proposed reaction models, it was found that the steps related to the generation of levoglucosenone (LGO) (by catalytic dehydration of levoglucosan (LG)) and acrolein (by breakdown of glycerol during CAFB burning—which was carried out through glycerol adsorption on a TiO_2_ surface in a the developed dehydration mechanism) represent rate-controlling steps, which are strongly controlled by applied heating rate. Isothermal predictions have shown that CAFB manifests very good long-term stability at 60 °C (which corresponds to storage in a sea shipping container), while at 200 °C, it shows a sudden loss in thermal stability, which is related to the physical properties of the sample.

## 1. Introduction

**Figure 1:**
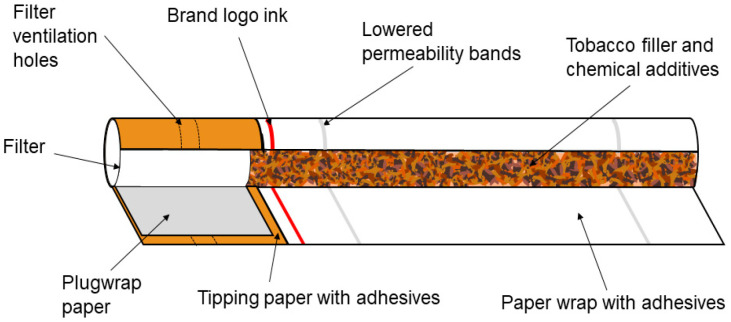
Components of the typical cigarette.

Cigarette butts are the most common garbage lying in city streets, restaurants, bus stops, parks, and other public places [1]. Although cigarette butts are small, they are considered harmful to the society and environment. Cigarette butts cause pollution as they are difficult to recycle and are often discarded; therefore, their collection and clean-up are difficult [2]. Cigarette butts are estimated to be the most discarded item in the world [3]. There are approximately 1 billion smokers worldwide, accounting for more than 6 trillion cigarettes a year, and more than 75% of cigarette butts are discarded in the open environment [4]. Discarded cigarette butts retain carcinogenic and toxic substances when littered in the open environment, which leach out to pose a threat via the contaminated soil, water, and biota [5]. Cigarette butts are also the most common form of plastic waste found along ocean coasts, threatening the quality of seawater and the survival of marine life. Therefore, the development of technology for the safe conversion of cigarette butts into usable energy offers significant benefits for both environmental protection and energy production. Cigarette butts represent the residuals remaining at the conclusion of the smoldering phase combustion, following the smoking of a cigarette. Factory-made (as ready-made) cigarettes have a relatively simple design with few components, which is shown in Figure 1.

They consist of a filler of cut tobacco that has been processed and mixed with chemical additives (additives increase the addictiveness of tobacco products). The filler is held in a porous paper wrap. Almost all cigarettes contain a filter at the mouthpiece, which is often wrapped in plug−wrap paper (an inner paper around the filter) and then tipping paper on the outside. Adhesives are used to fasten these papers. Inks are used to print names and logos on the paper or the tipping paper. Bands of lowered permeability are often added to the paper wrap to reduce the potential for starting a fire.

The tobacco, additives, papers, inks, and adhesives in cigarettes are made up of a wide range of chemicals, some of which are toxic and carcinogenic. When a cigarette is smoked, the tobacco filler, additives, and paper wrap, as well as the adhesives and ink on that paper, are burned. The components that remain unburned include the filter, tipping paper, plug−wrap paper, and the adhesives and inks used on these. The burned components undergo chemical reactions during burning that convert them into smoke and ash. During the production of smoke, many more toxic and carcinogenic chemicals are produced from these chemical reactions.

However, observing cigarette filters only, they are often made of plastic (cellulose acetate) fibers, paper, and/or charcoal. Namely, a single cigarette filter contains approximately 12,000 fibers of cellulose acetate (CA) (note: CA refers to any acetate ester of cellulose, usually cellulose diacetate (CDA)) [6]. These fibers consist of titanium dioxide (TiO_2_) and are connected together by a triacetin (TA) (glycerol triacetate) surfactant [7]. The large amounts of carbon atoms in the CA structure make it a good candidate to be used as a raw material for producing the porous carbon derivatives. Therefore, the primary component of cigarette filters (and cigarette butts as waste products after smoking) represents CA, and it is a desirable organic carbon source for conversion into useful liquid finished goods, using thermal cracking techniques, such as pyrolysis. Namely, pyrolysis has been successfully used for the decomposition of cellulose to furans, carboxylic acid, and aldehydes, as well as hydrocarbon-aromatics [8,9,10]. From the indicated research, the pyrolysis process represents an effective temperature-intensified procedure for recycling and reusing cigarette butt waste, in terms of their recovery and utilization. Therefore, the cigarette butts were directly pyrolyzed under different experimental conditions to produce cigarette-derived functional carbon, which has dual functional applications in super-capacitors and water pollution removal [11,12]. Nitrogen-doped cigarette-butt-derived carbon (N-CBDC) was prepared by continuous carbonization, activation, and the subsequent hydrothermal method and applied to electrode materials [13,14]. The production of the hydrochar by the hydrothermal carbonization of cigarette butts was found to be a significant place for accessing new and cost-effective fuel resources, while considering environmental benefits [15]. There is research that uses the green approach to recover the CA fiber from cigarette butts with filter fiber characterization protocols to evaluate the yarn-making capabilities of these fibers [16]. Furthermore, a high efficiency air filter based on ultrafine nanofiber, obtained from the waste of cigarette butts, was produced [17]. Likewise, the kinetic analysis of the pyrolysis process in cigarette butts and their components towards its energy recovery potential was found also to be significant [18,19].

It should be noted that the secondary component of cigarette filters—triacetin (TA), which is obtained from cigarette butts, represents the feedstock for low-cost and efficient biodiesel production [20]. Conventional diesel engines use fuel with 20% of biodiesel without difficulty, and many new engines can already use pure biodiesel. Whether used in its pure form or as a blend with conventional diesel, biodiesel is one of the best alternatives to fossil fuels and provides a solution to reduce emissions. The biggest drawback is the high production costs and potential pollution from biomass sources. An effective solution to reduce the price is mixing biodiesel with *triacetin*, which contributes to increasing its flammability. However, TA is usually produced through chemical reactions, and these processes generate toxins and other waste. Therefore, a way to reduce production costs was devised. Yousef et al. [20] have developed an environmentally friendly way of extracting TA from cigarette butts. The use or recycling of cigarette butts in this way reduces waste, puts it to sustainable use, and reduces the costs of TA production. This study experimentally proved that the pyrolysis process has great potential for the disposal of cigarette waste and can be used as a sustainable source of TA.

In summary, the review of all these studies actually has as its main goal the achievement of sustainable energy from waste, such as cigarette butt filters. On the other hand, the direct combustion of raw waste materials to provide heating energy is called thermal combustion, which is usually performed to generate steam and electricity. The main principle of gasification technology is producing synthetic fuel gas to be combusted for heat generation or used as turbine or engine fuel for electricity generation. The liquid fuel produced by the pyrolysis can be used as a fuel oil in static heating and applied for electricity generation. However, the valorization of cigarette butts for the production of value-added bio-fuels and chemicals through the combustion process, including reaction mechanism evaluation, is not studied enough in the scientific community. To the authors’ best knowledge, there are few papers related to the oxidative properties of smoked cigarette filters (SCFs) as a rich source of microfibers. Namely, the paper that can be highlighted belongs to De Fenzo et al. [21], where the authors evaluate the quality of recovered CA, which was characterized by thermogravimetric analysis (TGA) in an inert (N_2_) and oxidant (air) ambient and compared to unused CA. They used differential scanning calorimetry (DSC) analysis to define the glass transition temperature (*T_g_*) and possible crystallinity degree of the investigated samples. FTIR spectroscopy was used for delineation of the functional groups, after the cleaning process, and to determine if any difference is present in the recovered CA, with respect to the unused CA. In addition to these instrumental analyses, the authors [21] performed a standard kinetic analysis of the processes in both reaction atmospheres, using the standard Kissinger maximum-peak method and the Flynn–Wall–Ozawa isoconversional (model-free) method to calculate the activation energy (*E_a_*) values. However, this study did not provide any detailed information about the reaction mechanism of these processes but only general kinetic tip-offs. This especially applies to the mechanistic conclusions attached to the multiple-step process in the case of CA oxidation, and there is no information about the ignition and burnout properties of the extracted material (CA) from the cigarette butts.

The main goal of this paper is to investigate the combustion properties (through evaluation of the combustion characteristic parameters, such as ignition temperature, *T_i_* (the starting burning temperature); the maximum (peak) combustion rate temperature, *T_p_*; the maximum (peak) combustion rate, *R_p_*; and the burnout temperature, *T_b_*) and combustion performances (through evaluation of the different combustion indices) of cellulose ester fibrous bundles, separated from the used cigarette filters (cigarette butts). These analyses were conducted using simultaneous TG (thermogravimetry), DTG (derivative thermogravimetry), and DTA (differential thermal analysis) techniques, at various heating rates, in an air atmosphere. The obtained results provide insightful information on the evaluation of cellulose ester combustion. In this study, the mass spectrometry (MS) analysis was used for structural identification and diagnosis of which cellulose ester compound (cellulose diacetate (CDA) or cellulose triacetate (CTA)) is present in the fibrous bundles as testing samples. For that purpose, the matrix-assisted laser desorption/ionization–time-of-flight (MALDI-TOF) mass spectrometry technique was used. It should be pointed out that in this study, the modified procedure for preparing a fibrillary sample based on cellulose ester for MALDI-TOF measurements was exposed. In addition to the thermal and structural characterization of the investigated polymer material, a detailed kinetic analysis of the combustion process was carried out using two approaches: the isoconversional (model-free) and model-based methods. These approaches are strictly implemented in accordance with the ICTAC Kinetics Committee recommendations for the analysis of multi-step kinetics [22]. Therefore, the appropriate kinetic calculations were performed in order to obtain a complete scheme of the reaction mechanism that realistically reflects the degradation process of the studied polymer material in non-isothermal conditions. In addition to this, an isothermal prediction of the process was carried out in order to determine the thermal stability of the material under different temperature storage conditions. The results given in this research represent new data related to the combustion process of fibrous cellulose ester, mechanically separated from the used cigarette filters.

## 2. Materials and Methods

### 2.1. Materials

**Figure 2:**
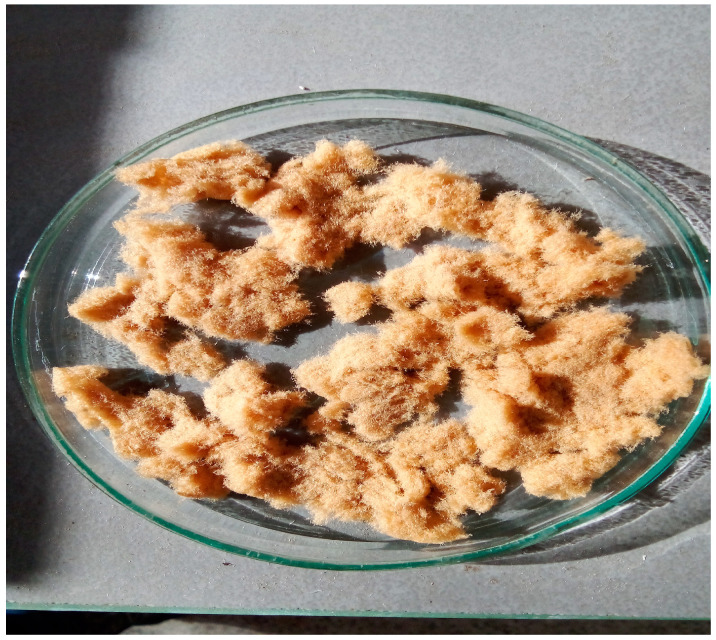
External appearance of the experimental sample examined in this study (CAFB sample).

The collected cigarette butts from the same manufacturer (the same cigarette brand from a USA Company, Philip Morris International, Chesterfield, MI, USA) were used as the initial feedstock. The cigarette butts were cleaned on the cardboard from residual ash and other visible “impurities” (referring to the unburnt tobacco that remained). After that, the used cigarette filters (previously unwrapped from the paper), primarily containing the CA fibers, are mechanically separated from the remaining parts of the cigarette butts. The separation of the filters from the ash, the remaining tobacco, and the paper was carried out by hand. It should be noted that today’s cigarette filters contain approximately 95% CA (the so-called “plug” of acetate cellulose filter tow), and CA tow fibers are thinner than sewing thread, white, and in production, they are tightly packed to create a filter. The cleaned filters were then opened by cutting them lengthwise with a razor, revealing a fibrous mass, i.e., fibrous bundles. After this procedure, spreading apart the matrix, the fibrous sampling material was isolated. Thereafter, the fibrils were chopped with scissors into smaller pieces, they were put in the mill in order to grind, and thus, the final sample for the measurements was prepared. The material used for the experimental measurements is shown in Figure 2.

It should be noted that in addition to the fibrillary polymer material based on cellulose ester, these filters contain titanium dioxide (TiO_2_) as a delustering agent and triacetin (TA) as a plasticizer [12]. The last, TA, is the most commonly used plasticizer for CA filters (the role of the TA is to bond the neighboring fibers to each other). To obtain a sufficient hardness, the target values for triacetin vary typically between approximately 6% and 9% of the total filter weight [23]. The mentioned TiO_2_ has a role as a “whitening” agent for the filter material. Namely, TiO_2_ is bound to the CA of the filters. In most cases, the fibers are usually in a Y-shape form and contain TiO_2_. TA is used for the solidification of the acetyl cellulose fibers in the manufacture of cigarette filters; therefore, TA is present in our experimental samples. In the following text, our sample is marked as the *cellulose acetate fibrous bundle* (CAFB).

### 2.2. MALDI-TOF Characterization of CAFB Sample

The sample preparation is an important step in the MALDI analysis. The density and morphology of the sample/matrix crystals directly affect the resulting mass accuracy, the resolution, and the reproducibility of the analysis. The careful selection of matrix and ionizing agents, the removal of impurities, and proper sample preparation are essential for accurate and reliable results [24] (pp. 1–476). However, it should be noted that the matrix can sometimes cause problems, such as signal suppression, uneven crystallization, analyte/matrix fragmentation, etc. Therefore, careful optimization of the sample preparation procedure is crucial to overcome these problems and ensure a reliable MALDI analytical performance.

The established conventional method for the sample preparation and application in the MALDI-TOF analysis involves dissolving the test substance in a suitable solvent and transferring it to the MALDI plate in the form of droplets. A suitable matrix is then prepared and applied over the previously applied sample to support the ionization of the tested sample. The conventional methods for the sample preparation in MALDI-TOF analysis usually differ in the way in which the matrix solution is combined with the analyte solution. The dry droplet method, as well as the sandwich and thin layer methods, are commonly used.

For the experimental investigation of the CAFB sample in this study, a different, unconventional method of sample application was used. Following the approach of Skelton et al. [25] for the investigation of poorly soluble polymers, in which the matrix was mixed with the polymers and pressed into pellets, a similar principle was applied here.

A double-sided adhesive tape was first stuck to the MALDI plate, onto which finely chopped segments of the CAFB sample were carefully placed with tweezers. This prepared sample was then introduced into the ionization chamber of the MALDI device, where the sample was ionized with an ultraviolet nitrogen laser (wavelength 337 nm, pulse width 3 ns, and repetition rates of 20.00 Hz) and a time-of-flight (TOF) analyzer. The accelerating voltage was 25 kV, with 78% grid voltage and an extraction delay time of 150 ns. The laser intensity was set to 3400 arbitrary units, and the number of laser shots per spectrum was 250.

### 2.3. Simultaneous TG-DTG-DTA Measurements of CAFB Sample in an Air Atmosphere

The SETARAM SETSYS Evolution 1750 instrument (SETARAM Instrumentation, 7 Rue de l’Oratoire, 69300 Caluire-et-Cuire, France) was used for non-isothermal thermogravimetric analysis (TGA) and differential thermal analysis (DTA) of the CAFB sample, in an air atmosphere, at four different heating rates: *β* = 5.1, 10.5, 21.5, and 32.8 K/min. The samples were heated according to the linear heating program. To minimize the effects of mass and heat transfer limitations, approximately Δ*m*_(sample)_ = 6.0 ± 0.05 mg of the samples were loaded into an alumina crucible. The recording at each heating rate was performed at an air-flow rate of *ϕ* = 20 mL/min in the temperature range from ambient temperature up to 700 °C. Duplicate non-isothermal runs were performed under similar conditions, and the data were found to overlap with each other (including the control measurement for each heating rate, with approximately the same sample mass), indicating a satisfactory reproducibility. This was implemented in order to minimize possible errors during the measurement. The experimental TGA-DTA test was carried out according to comprehensive calibration and measurement procedures, which precede the sample measurement. It is defined by an equipment manufacturer in order to handle the reproducibility and productivity of the results. The calibration procedure was performed according to the manufacturer’s instructions and the measurements with empty crucibles in order to correct the measured signals and handle the mass balance deviations. The operating system of the device for data handling is capable of providing a construction of the derivative thermogravimetric curve (DTG) at each heating rate used.

#### 2.3.1. Combustion Characteristic Temperatures

**Figure 3:**
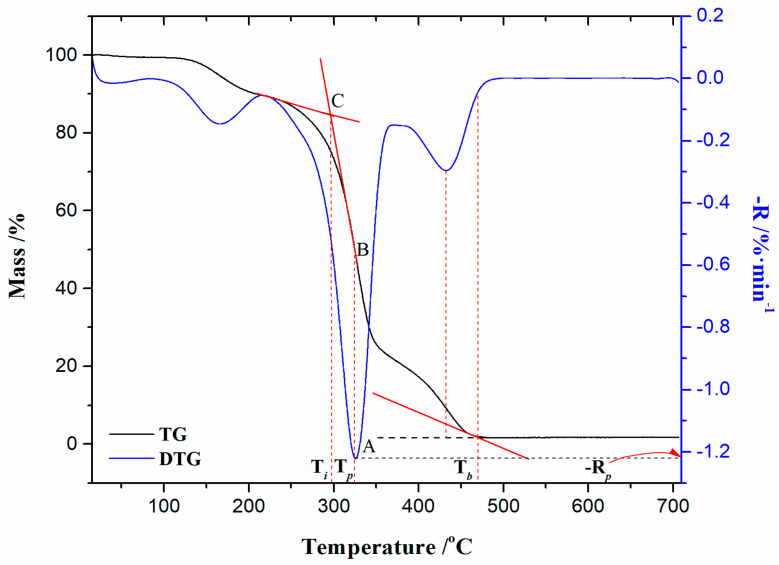
Schematic diagram of TG and DTG curves for application of tangent method in the evaluation of basic combustion parameters: *T_i_*—ignition temperature, *T_p_*—maximum (peak) temperature, and *T_b_*—burnout temperature; point A is the point at which a vertical line from the main DTG peak (highest value) crosses the TG curve; point B is the position on the TG curve at which the vertical line from the “second” peak of DTG curve crosses the TG curve; point C is the point where a tangent at the TG curve from the left crosses the tangent drawn through the point B, and the corresponding temperature at the intersection of two lines is recognized as *T_i_*; the temperature *T_b_* is determined as the point of crossing between the tangent drawn on the TG curve and the horizontal line, drawn from the last part of the TG curve. The symbol “*R_p_*” represents the maximum combustion rate (%·min^−1^).

The TG-DTG and DTA curves allowed us to determine the thermal behavior of the tested sample during the heating in an air atmosphere at a fixed atmosphere. The main parameters related to the specific temperatures can be identified if the properly scanned TG and DTG curves were obtained, and they are as follows: the ignition temperature (*T_i_*), the maximum (peak) temperature (*T_p_*), and the burnout temperature (*T_b_*). These basic combustion parameters were identified from TG-DTG curves, whereby their determination was made by the TG-DTG tangent method [26,27], as shown in Figure 3.

Considering the above analysis, we can look at our sample as a combustible polymer in a gaseous oxidizer flow atmosphere (air) with the capability of a high-performance thermoplastic polymer. Namely, the ignition temperature (*T_i_*) has been investigated under radiative heating for different polymers by several authors [28,29] (pp. 234–352) and is generally reported to be practically independent from the imposed heat flux for non-charring polymers, whereas decreasing with an increase in the irradiance was reported for charring polymers, such as natural cellulosic polymers [29] (pp. 234–352). Since, in this study, the combustion measurements were performed at a micro-scale with milligram samples, the appropriate definitions for *T_i_*, *T_p_*, and *T_b_*, must be presented. *T_i_* represents the temperature at which the material starts burning, *T_p_* is the temperature that corresponds to the DTG peak temperature (R*_p_* is the maximum combustion rate—the rate of the loss is the maximum at DTG peak—Figure 3), and *T_b_* represents the temperature that corresponds to the mass loss of the sample that exceeds 99%, indicating that the investigated material has stopped burning [30].

#### 2.3.2. Combustion Performance Indices

To evaluate the combustion performance of the CAFB sample, the combustion indices, including the ignition index (*D_i_*) [%·min^−3^], the burnout index (*D_b_*) [%·min^−4^], flammability index (*C*) [%·°C^−2^·min^−1^], comprehensive combustion index (*S*) [%^2^·°C^−3^·min^−2^], and the intensity index of the combustion process (*H_f_*) [°C] were determined, according to well-established expressions [31,32], through Equations (1)–(5):

(1) $$D_{i}=\frac{\left(-R_{p}\right)}{t_{i}\cdot t_{p}}$$

(2) $$D_{b}=\frac{\left(-R_{p}\right)}{∆t_{1/2}\cdot t_{p}\cdot t_{b}},$$

(3) $$C=\frac{\left(-R_{p}\right)}{T_{i}^{2}}$$

(4) $$S=\frac{\left(-R_{p}\right)\cdot \left(-R_{v}\right)}{T_{i}^{2}\cdot \;T_{b}},$$

(5) $$H_{f}=T_{p}\cdot ln\left[\frac{{∆T}_{1/2}}{\left(-R_{p}\right)}\right]\cdot 10^{-3},$$

where *R_p_* is the maximum combustion rate (%·min^−1^), *t_i_* is the ignition time (min), *t_p_* is the corresponding time to the *T_p_*−value (*R_p_*-value) (min), *t_b_* is the burnout time (min), Δ*t*_1/2_ is the time range of *R*/*R_p_* = 0.5 (min), *R_v_* is the mean combustion rate (%·min^−1^), then Δ*T*_1/2_ is the temperature range of *R*/*R_p_* = 0.5 (°C), and *T_p_* is the peak temperature (°C).

### 2.4. Kinetic Analysis

Under the fixed heating rate of *β* (*β* = d*T*/d*t*), the reaction rate of the combustion of the solid material under non-isothermal conditions can be defined by Equation (6):

(6) $$\beta \cdot \frac{d\alpha }{dT}\equiv \frac{d\alpha }{dt}=A\cdot exp\left(-\frac{E_{a}}{RT}\right)\cdot f\left(\alpha \right),$$

where d*α*/d*t* represents the rate of the combustion process (s^−1^); *A* is the pre-exponential factor (s^−1^); *E_a_* is the activation energy (J/mol); *R* is the universal gas constant (8.314 J·mol^−1^·K^−1^); *T* is the absolute temperature (K); *f*(*α*) is the differential form of the reaction model; while *α* is the conversion (the extent of reaction; dimensionless), expressed as *α* = (*m_o_* − *m*)/(*m_o_* − *m_∞_*), where *m_o_* and *m_∞_* are the original (initial) and ultimate mass of the sample, respectively; and *m* is the actual mass of the sample at instantaneous temperature (*T*) and time (*t*). The parameters *A*, *E_a_,* and *f*(*α*) represent the kinetic triplet, which are to be determined during the kinetic analysis of the investigated combustion process.

The integral form of Equation (6) leads to the integration of the Arrhenius equation, giving Equation (7):

(7) $$g\left(\alpha \right)=\int_{0}^{\alpha }\frac{d\alpha }{f\left(\alpha \right)}=\frac{A_{\alpha }}{\beta }\int_{0}^{T_{\alpha }}exp\left(-\frac{E_{a,\alpha }}{RT_{\alpha }}\right)dT_{\alpha }≅\frac{A_{\alpha }}{\beta }\cdot J\left(E_{a,\alpha },T_{\alpha }\right),\;$$

where *g*(*α*) is the integral form of *f*(*α*), *J*(*E_a_*_,*α*_,*T_α_*) is the temperature integral, while *T_α_*, *A_α_* and *E_a_*_,*α*_ represent the temperature at given conversion (*α*), the pre-exponential factor, and the activation energy values at considered conversion (*α*), respectively. Since *f*(*α*) is independent from the temperature and heating rate, this is likewise for its integral term. Note that the derivation of the above equation involves an assumption that *E_a_* must be a constant with respect to conversion; the temperature integral *J*(*E_a_*_,*α*_,*T_α_*) has no analytical solution, but many approximations exist with the aim of solving this integral through approximated procedures [33,34].

#### 2.4.1. Isoconversional (Model-Free) Analysis: Friedman (FR), Vyazovkin (VY), and Numerical Optimization (NM) Methods

Model-free analysis allows for the determination of the activation energy in a reaction process without assuming a kinetic model for the process. Furthermore, the reaction type is usually not required to calculate the activation energy. However, it is not possible to determine the number of reaction steps, their contribution to the total effect, or the order in which they occur. These methods calculate values of the logarithm of the pre-exponential factor (*logA*) for the assumed function *f*(*α*), usually based on the first-order kinetics (*f*(*α*) = 1 − *α*).

Model-free analysis is based on two main assumptions:

(a) the reaction can be described by only one kinetic equation for the extent of the reaction, *α*, as follows:

(8) $$\frac{d\alpha }{dt}=A\left(\alpha \right)\cdot f\left(\alpha \right)\cdot exp\left(\frac{-E_{a}\left(\alpha \right)}{RT}\right),$$

where *E_a_*(*α*) is the activation energy depending on the conversion, *α*, and *A*(*α*) is the conversion−dependent pre-exponential factor. The activation energy (*E_a_*) is calculated without any assumption about reaction type, but the pre-exponential factor (*A*) can be found only under the assumption about the reaction type.

(b) The reaction rate at a constant value of conversion (*α* = *const*.) is only a function of temperature.

The Friedman (FR) isoconversional (model-free) method [35] relies on a differential solution of Equation (6), with the assumption that the chemistry of the combustion process depends only on the rate of mass loss and is independent from the temperature. The Friedman relation can be derived by taking logarithms of both sides of Equation (6), which takes the final form, as follows:

(9) $$ln{\left(\frac{d\alpha }{dt}\right)}_{\alpha ,i}=ln{\left[A_{\alpha }\cdot f\left(\alpha \right)\right]}_{i}-\frac{E_{a,\alpha }}{RT_{\alpha ,i}}.$$

where subscript “*i*” designates the *i*-th value of the used heating rate (*β_i_*), while *T_α_*_,*i*_ represents temperature at which the given conversion (*α*) is reached at the corresponding *β_i_*. The activation energy (*E_a,α_*) at *α* = *const*. can be determined from the slope of the isoconversional line at a given value of *α*. The linear plot of *ln*(dα/d*t*)*_α,i_* against 1/*T_α,i_* is generated for the different heating rates, and the activation energy is determined from *slope* = −*E_a,α_*/*R*.

The most accurate model-free method is the advanced integral method presented by Vyazovkin [36]. In this method (Vyazovkin (VY)), the activation energy is then obtained for each value of α at different temperatures, *T_i_*(*t*), by minimizing the function, *Φ*(*E_a_*), as

(10) $$\Phi \left(E_{a}\right)=\sum_{i=1}^{n}\sum_{j\neq i}^{n}\frac{J\left(E_{a,\alpha },T_{\alpha ,i}\right)\cdot \beta_{j}}{J\left(E_{a,\alpha },T_{\alpha ,j}\right)\cdot \beta_{i}}=min.$$

(11) $$J\left[E_{a,\alpha },T_{\alpha ,i}\left(t_{\alpha }\right)\right]=\int_{t_{\alpha -∆\alpha }}^{t_{\alpha }}exp\left(-\frac{E_{a,\alpha }}{RT_{\alpha ,i}\left(t_{\alpha }\right)}\right)dt.$$

In the above equations, *E_a,α_* and *T_α_* are the activation energy and the temperature at the conversion *α*, respectively, obtained from independent experimental runs *i* and *j* and performed at different heating rates, *β*’s. The integral is numerically evaluated by using the trapezoidal rule and the uniform grid spacing, which is continually decreased until a difference in the integral values smaller than 10^−6^ between consecutive interactions is obtained. This minimization can be carried out at the different values of *α* to obtain the related activation energy (*E_a_*). A similar procedure applies to the calculation of *logA* values.

The numerical optimization (NM) method represents the model-free method using the non-linear least square optimization. The numerical method searches the optimal functions such as *E_a_*(*α*) and *logA*(*α*) in order to obtain the best fit for the conversion (*T*, *t*). The numerical method is based on the results of the analytical Friedman method (it is often called the modified Friedman method). Results of the Friedman method (curves *E_a_*(α) and *A*(α)) are optimized numerically in order to achieve the better fit between the experimental and simulated curves.

The calculation of *logA* is implemented for the first order reaction. The function for optimization is the sum of the squares of deviations between the measured value, *Conversion-experimental* (*T*), and the calculated value, *Conversion-simulated* (*T*). This sum is calculated over all the curves and over all the points in each curve, as follows:

(12) $$\Omega =\sum_{Curves}\sum_{Points}{\left[\alpha {\left(T\right)}_{i}^{calc}-\alpha {\left(T\right)}_{i}^{exp}\right]}^{2},$$

where *α*(*T*)*_i_^calc^* and *α*(*T*)*_i_^exp^* represent the calculated and experimental conversion values (furthermore, this procedure *can be implemented on TGA-signals*/mass-loss–temperature signals) for the considered *i*-th heating rate used. The numerical method searches the numerical values *E_a_*(*α*) and *logA*(*α*), which minimize the function, *Ω*. Internally, each point of curves *E_a_*(*α*) and *A*(*α*) is a subject of the small changes, and for each change, the sum of squares of residuals is checked: is it better or worse than before? If better, then the new point in the *E_a_*(α) or *A*(*α*) is saved. The iterations are repeated until no any numerical improvements happen.

The advantage of this numerical optimization is reflected in the fact that it can be applied to the multiple-step reactions with evaluation of each reaction point at the various heating rates.

For all the presented conversion-dependent methods, the Kinetics Neo computational kinetics software (Version 2.7.0.11; Build date: 21 January 2024) was used. This software, for application of the Friedman (FR) method, instead of the “*ln*” scale, uses the “*log*” scale data where it normally operates. Considering model-free methods, the kinetic parameters are determined using the points at the same conversion, between *α* = 0.01 (*α* = 1%) to *α* = 0.99 (α = 99%), with the conversion step increment of Δ*α* = 0.01, from measurements at four different heating rates.

#### 2.4.2. Model-Based Analysis

The model-based kinetic analysis represents the procedure for the complex chemical processes that contain the individual reaction steps, where each step can be individually connected to another reaction step (consecutive, competitive, independent, etc.) in order to build a kinetic model of the complex process under investigation. The model-based kinetic approach describes the reaction rate of multi-step chemical reactions by a system (or the set) of kinetic equations, where each reaction step has its own kinetic equation and kinetic triplet, containing activation energy (*E*), the pre-exponential factor (*A*), as well as the reaction type (Appendix A—Table A1). The applied software (Kinetics Neo; Product version: 2.7.0.11, Build date: 29 January 2024) uses the multivariate non-linear regression method (MVarNLRM) to resolve the concentration equations in the multi-stage process. The type of the *f*(*α*) function (Appendix A—Table A1) depends on the nature of the process and is usually selected a priori. For the user’s convenience, the notation of parameters and variables in Table A1 is the same as in the Kinetics Neo software. The software uses the *p* parameter, which corresponds to the conversion (*p* = *α*), and the parameter *e* = 1 − *α* (the remaining fraction unreacted). The model-based analysis performed by the software assumes that the investigated process may comprise several elementary reaction steps, which can be a series of consecutive reactions.

The reaction rate for the individual reaction steps can be expressed through Equation (13) [37,38]:

(13) $${Reaction\;rate}_{j}=A_{j}\cdot f_{j}\left(e_{j},p_{j}\right)\cdot exp\left(-\frac{E_{j}}{RT}\right),$$

$${\left(\frac{d\alpha }{dt}\right)}_{j}={\left[\frac{d\left(a_{j}\;\to \;b_{j}\right)}{dt}\right]}_{j}=A_{j}\cdot exp\left(-\frac{E_{j}}{RT}\right)\cdot f_{j}\left(a_{j},b_{j}\right),$$

where *f_j_*(*e_j_*,*p_j_*) represents the function of the reaction type, *e_j_* is the initial reactant concentration, *p_j_* is the product concentration, *A_j_* is the pre-exponential factor, *E_j_* is the activation energy, while the *j* represents the number of specific reaction steps ((d*α*/d*t*)*_j_* is the reaction rate for the step with the number *j*, *a_j_* is the concentration of the initial reactant, *b_j_* is the concentration of the product). This approach takes into account certain assumptions, such as

(a) the process has to consist of a number of reaction steps, and for each of these steps, the reaction rate can be described by Equation (13), depending on the concentration of the initial reactant, *e_j_*; the concentration of the product, *p_j_*; the pre-exponential factor, *A_j_*; and the activation energy, *E_j_*; (b) the relevant factors, including activation energy, pre-exponential factor, the order of reaction, and the reaction type for each step, are considered to be constant (assumed to be constant during the reaction progress, for every individual reaction step), and (c) it is assumed that the thermo-analytical signal equals the sum of the signals of the single reaction steps, while the effect of each step is calculated by multi-plying the reaction rate with the mass loss of this step, expressed through Equation (14):

(14) $$m=m_{o}-\Delta m\cdot \left[\sum_{j=1}^{n}{Contribution}_{j}\int {\left(\frac{d\left(x_{j}\;\to \;y_{j}\right)}{dt}\right)}_{j}dt\right]$$

where *m* is the mass, *m_o_* is the initial mass, Δ*m* is the total mass change, while (*x_j_* − *y_j_*) represents the heat flow on the reaction path from the reactant “*x*” to the product “*y*”, and “*Contribution_j_*” corresponds to the contribution of the “*j*” reaction step to the overall heat flow. For the model-based method, the change in kinetic mechanism is simulated by several reaction steps, with their own kinetic triplets (*A_j_*, *E_j_*, and *f_j_*), ranked in a specific reaction order through a constructed mechanistic (reaction) scheme, which has special marking in actual software (the scheme code designation), depending on whether branching exists or not.

This kinetic approach allows for the accurate estimation of the number of the present (actual) reaction steps, their contribution to the total effect of the reaction, and the corresponding reaction orders (or kinetic exponents) for each of the considered reaction steps. Therefore, the result of the model-based analysis provides information about the reaction mechanism, the form of equations for the elementary steps, and the values of the kinetic triplets [*A*, *E*, *f*(*α*)] for these steps. The considered model-based kinetic analysis was performed by Kinetics Neo software (Version 2.7.0.11; Build date: 29 January 2024) and can be based on the models, which include several process steps in which the individual steps are linked as the independent, parallel, competing, or following reactions. Each model-selected reaction type for each step has some unknown kinetic parameters, such as activation energy, pre-exponential factor, and reaction order, as well as the contribution of each step to the overall process. All unknown parameters can be found from the fit of the measured data with the simulated curves. For this procedure, the software uses multivariate non-linear regression (MVNLR) [38]. The statistical comparison of the fit for the different kinetic models allows us to select an appropriate kinetic model with a corresponding set of kinetic parameters. The fit (the final) results present the agreement between the experimental and simulated curves for the TG-signal, conversion, the conversion rate, or the concentration of all reactants. The unique flexible model designer in the Kinetics Neo software offers the possibility of the visual design for the kinetic models with an unlimited number of reaction steps, connecting in any combination. Thus, the simulated reaction step can be visually moved to the corresponding step on the experimental curve. Then, the parameters of this step can be optimized.

#### 2.4.3. Simulation Tests—Isothermal Predictions

The ability of the presented kinetic analysis is to predict the effect of thermal aging on the investigated CAFB sample when the investigated material is subjected to the isothermal accelerated aging tests. Namely, in this work, the simulation test was performed under isothermal conditions, which include analysis of the material’s long-term stability. Therefore, in order to evaluate our tested sample with regard to its long-term stability, isothermal predictions at different temperatures were calculated over a time period of 12 months (1 year). Starting from *T* = 0 °C (freezing conditions), across *T* = 20 °C (the ambient storage conditions), and *T* = 60 °C (the temperature that corresponds to a maximum temperature for the sea shipping container), until *T* = 200 °C (highly demanding temperature storage conditions), the conducted predictions give a comprehensive insight into the thermal stability of the investigated polymer material, ranging from frozen storage to high-temperature stress conditions. All the simulations were based on data obtained from the applied kinetic methods/models.

## 3. Results and Discussion

### 3.1. Displaying of MALDI-TOF Results for Modified Polymer Characterization—Identification of the Type of Cellulose Ester in Tested CAFB Sample

CA belongs to the group of modified polymers. CA is obtained as the result of the acetylation reaction of cellulose with acetic anhydride in glacial acetic acid as the medium of reaction. The process occurs in the presence of zinc(II) chloride or sulfuric acid acting as the catalyst. Currently, the synthetic polymeric materials containing the built-in cellulose acetate into the main chain are the aim of the studies [39]. Materials containing CA are used for the production of various elements that adversely affect the environment, e.g., cigarette filters [39]. In this context, in our study, a new approach to the preparation of the fibrous polymer material based on CA was applied, which is described previously.

**Figure 4:**
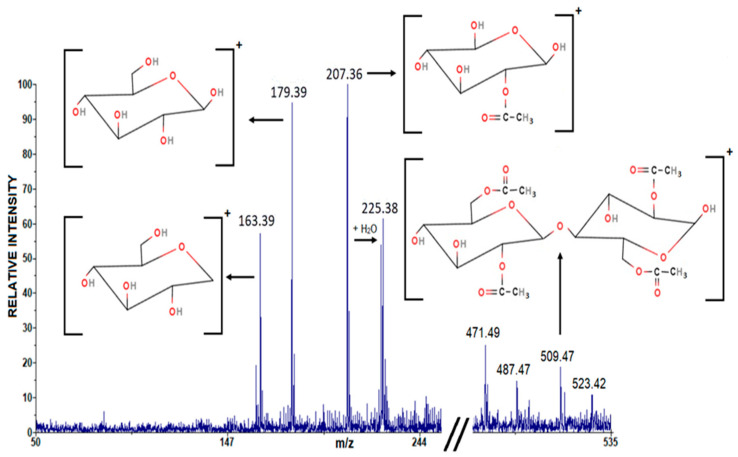
MALDI-TOF-MS spectrum of CAFB sample, where the existence of corresponding ion species (chemical fragments) is also indicated.

A selected portion of the positive mode LDI mass spectrum of the CAFB sample contains several groups of signals identified as follows: *m*/*z* 163.39 [C_6_H_11_O_5_]^+^ (calcd *m*/*z* 163.15), *m*/*z* 173.39 [C_6_H_11_O_6_]^+^ (calcd *m*/*z* 179.15), *m*/*z* 207.36 [C_7_H_11_O_7_]^+^ (calcd *m*/*z* 207.16), and *m*/*z* 509.47 [C_20_H_29_O_15_]^+^ (calcd *m*/*z* 509.44) (Figure 4).

The peaks at masses 163.39, 179.39, and 207.36 represent different fragments of cellulose diacetate (CDA). Mass 163.39 corresponds to the glucose ion without the OH group, mass 179.39 corresponds to the glucose ion without the H atom, while mass 207.36 represents a fragment of the monomer unit of cellulose diacetate (CDA) in which the bond between the fifth and sixth carbon atoms has been broken, so that the ester group has been replaced by an OH group. The peak at mass 509.47 represents the parent ion of cellulose diacetate (CDA). Table 1 shows the experimentally determined values of the cellulose diacetate (CDA) signal and its fragments in the positive LDI mode, as well as their theoretical values.

Previously, Kunusa et al. [40] and Li et al. [41] used MALDI-TOF and ESI-QTOF to study microcrystalline cellulose and determine the cellulose oligomer fractions with different molecular weight distributions. They detected cellulose oligomers with sodium or/and potassium adducts.

Since we took a different approach to analyzing the CAFB sample in this study by adhering fibrils from virgined feedstock (e.g., the cigarette butt filters) directly onto a double-sided adhesive tape, it was possible to detect the parent ion of the CDA (cellulose diacetate) without forming adducts with sodium/potassium. As far as we know, this is the first time that CDA has been detected using the mass spectrometry (MS) technique (LDI).

Considering the small amounts present in the investigated sample, and by applying an unconventional type of sample preparation (finely chopped segments of CAFB taped to the MALDI plate, without dissolving the sample in a suitable solvent and without the application of a matrix), TA and TiO_2_ remained undetectable, falling below the detection threshold for this type of applied spectroscopic technique.

### 3.2. TG-DTG-DTA Analysis of CAFB Combustion Process under Non-Isothermal Conditions

Thermal analysis techniques are used for the purpose of testing the pyrolytic properties of various polymers, but the thermal characteristics of cellulose derivatives in oxidative conditions are important from the aspect of safety countermeasures, as well as for obtaining the value-added products. In the first case, it is important to distinguish the thermal history of the process, which is monitored by this instrumental technique, because it can be distinguished from the stable thermal degradation process (where the DTG/DTG curve shows a symmetrical bell-shaped pattern) or the combustion process and/or thermal runaway (the process is characterized by the appearance of a DTG/DTA curve that can have a sharp shape, like a needle). This is significant because cellulose ester has a wide range of industrial sector applications (packing, textile, plastic, photo, surface coating, tobacco) where its behavior in an air atmosphere is very important, from storage to other important demands for further treatment. This study is focused on CA filters as an important substrate for the production of value-added chemicals via the combustion process (the second case of application, where the reactivity of the cellulose derivative can be tested under oxygenated conditions).

**Figure 5:**
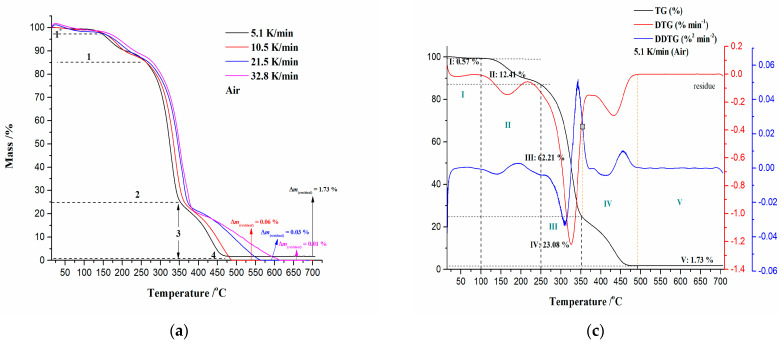

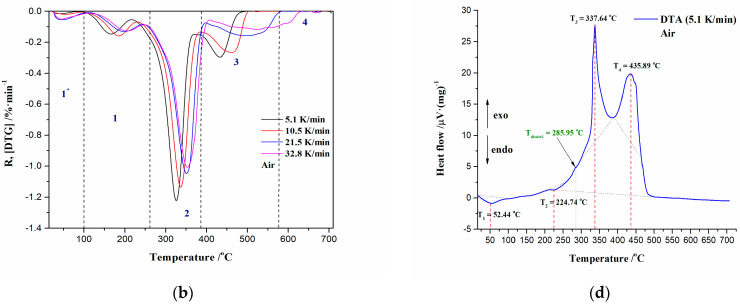
Thermo-analytical curves of CAFB combustion process under non-isothermal conditions: (**a**) TG curves of CAFB sample recorded at four different heating rates (5.1, 10.5, 21.5, and 32.8 K/min) in an air atmosphere (the main process stages are indicated by marks “1*, 1, 2, 3, and 4”; the values of residual mass were also indicated), (**b**) DTG curves (R/%·min^−1^) of CAFB sample recorded at four different heating rates (5.1, 10.5, 21.5, and 32.8 K/min) in an air atmosphere (the main process stages are indicated by marks “1*, 1, 2, 3, and 4”), (**c**) Simultaneous display of TG, DTG, and DDTG (derivative DTG) curves for CAFB sample recorded at the heating rate of β = 5.1 K/min in an air atmosphere with established mass losses regarding each reaction process stage (I, II, III, IV, and V), and (**d**) differential thermal analysis (DTA) curve for CAFB sample at the heating rate of β = 5.1 K/min in an air atmosphere with indicated characteristic process temperatures (T_1_, T_2_, T_deacet._, T_3_, and T_4_), attributed to the appropriate thermal effects.

Figure 5a–d show TG, DTG, simultaneous TG-DTG-DDTG (second derivative of TG curve), and DTA curves for the non-isothermal combustion process of the CAFB sample. Thermo-analytical curves for the considered process, shown in Figure 5c,d, are presented at a heating rate of β = 5.1 K/min.

Observing the appearance of the TG curves for the CAFB sample (Figure 5a), it can be concluded that the combustion process contains several reaction stages, suggesting its kinetic complexity. There are several reaction stages during combustion, as: Stage 1* (Figure 5a)—can be attributed to the removal of water present in the CAFB sample, which spans between RT (room temperature) and 100/150 °C, and it is characterized with very small mass loss (~0.57%) (Figure 5c). For this stage, the endothermic event identified in the DTA curve occurs at a temperature of 52.44 °C (temperature T_1_ in Figure 5d). This reaction stage can be clearly seen in the presented DTG curves (Figure 5b); Stage 1 (Figure 5a)—it was characterized by the removal of the plasticizer from the polymer matrix (the plasticizer volatilization).

The observed stage indicates a greater sample mass loss (~12.41%) than the previous one (Figure 5c). The degradation process of the plasticizer (TA) takes place with a continuous shift of the DTG peak towards a higher temperature with an increase in the heating rate (Figure 5b). The intensity of the DTG peaks vary with β’s, where higher degradation rates are achieved at lower heating rates (5.1 K/min and 10.5 K/min). It should be noted that this reaction is characterized by a small endothermic effect, which occurs at approximately ≈ 170 °C (this event is observed in the DTA curve, but it was not marked) (Figure 5d). The current process stage takes place in the temperature region, approx. between 100 °C and 225/250 °C (Figure 5a–c). It should be emphasized that in addition to the plasticizer degradation reaction, there is a release of the compounds that were trapped in the fibrous bundles during cigarette (tobacco) use [42], and they were classified as volatile organic compounds (VOCs).

Stage 2 (Figure 5a) is characterized by the greatest sample mass loss (~62.21%) (Figure 5c), which can be attributed to the rapid cellulose diacetate (CDA) oxidative degradation. This process can be seen by means of a strong exothermic effect, with a DTA peak located at T_3_ = 337.64 °C (Figure 5d). The reaction atmosphere has a powerful influence on the thermal degradation of cellulose and its derivatives. Namely, the cellulose is more stable in an inert atmosphere (nitrogen) than in an oxidative (air) atmosphere, because the oxygen can play the role of a catalyst in accelerating the degradation process of cellulose [43]. However, compared to the nitrogen and air atmospheres, the cellulose is the least thermally stable in a pure oxygen flow. Cellulose and its esters have much higher values of kinetic parameters (E_a_ and logA) in pure oxygen, than in air or nitrogen [43].

In the case of cellulose esters, the thermal stability can be guided by the degree of substitution (DS), where CA with DS = 2.5 is known as cellulose diacetate (CDA) [44]. Regarding this information, if we go back to Figure 5d, the endothermic peak located at T_2_ = 224.74 °C is associated with the melting temperature (T_m_) point of the CAFB sample. This melting temperature is slightly lower than the melting temperature range for cellulose acetate (CA) (230–300 °C), but truly, the obtained T_m_ is the characteristic for fibrous cellulose with the extent of modification (DS) of DS ≈ 2.5 (CDA) [45,46]. After the temperature T_2_ (=T_m_), there is another endotherm situated at a temperature of 285.95 °C, and this can be attributed to the deacetylation reaction (T_deacet._) (Figure 5d). Taking into account all the events so far, the melting of CAFB and the subsequent deacetylation take place in the temperature regions, which include process stages in a transition manner, as “1”→“2” (Figure 5b) and “II”→“III” (Figure 5c). The group of DTG peaks in the temperature range of ΔT = 265–390 °C (Figure 5b) belongs to the vigorous and sharp exotherm presented in Figure 5d, and this is due to the thermal degradation of the cellulose fibers [47]. Namely, the additional “second” group of DTG-peaks appearing approx. between 400 °C and 500 °C have a tendency to shift towards higher temperatures with the increase in the heating rate (Figure 5b). Namely, this behavior is typical for cellulose esters with higher DS [47], such as our identified CDA in the CAFB sample. Cellulose esters start to degrade at temperatures substantially lower than cellulose, but with higher DS, their thermal stability can be improved (Figure 5b). This improvement in thermal stability can be explained by the formation of new-ordered structures in the substituted regions. Namely, the exothermic peak (T_3_) following the endothermic peak at T_deacet._ (Figure 5d) is typical for cellulose ester, where observations linked with the recorded DTG and DDTG trails (Figure 5c) within process zones “III” and “IV” (for the last one, the sample mass loss was amounted 23.08%) can suggest the crosslinking reactions that may occur during the oxidative degradation [47]. The reactions that take place within both combustion zones, i.e., in “III” and “IV”, are obviously complex, involving fragmentation of the obtained products after thermo-oxidative degradation (it may involve the oxidative cleavage of glycosidic bonds, releasing of carbon dioxide (CO_2_) and water, primary and secondary reaction products, as well as other compounds that readily generate free radicals, which probably contribute in a chain radical mechanism) and then their combustion, as well as the char oxidation reactions. The process of char oxidation is manifested by a broad exothermic peak in Figure 5d, positioned at temperature T_4_ (=435.89 °C). Above 500 °C, the CAFB sample shows a progressive reduction in the solid residue with an increase in the heating rate (Figure 5a). As can be seen, the CAFB sample does not combust without residue, which suggests that there are unburned compounds that probably contain incorporated metal traces (originating from harmful species left in the filters after smoking).

It should be noted that the pronounced glass transition temperature (T_g_) of CAFB was not detected, which occurs in the T-interval of 120–180 °C [48], probably due to the disturbance of other ingredients in the CAFB sample.

#### 3.2.1. Ignition/Burnout Characteristics and Combustion Performances of CAFB Sample

Table 2 lists the values of the obtained characteristic combustion parameters (T_i_, T_p_, T_b_, and R_p_) and the combustion performance indices (D_i_, D_b_, C, S, and H_f_) for the CAFB sample at different heating rates (β = 5.1, 10.5, 21.5, and 32.8 K/min).

From the results presented in Table 2, it can be seen that the ignition temperature (T_i_) increases with the increase in the heating rate. This means that a tested sample with an increase in β undergoes a longer initial pyrolysis stage, which obeys the larger mass loss before starting conflagration. For instance, the mass loss at T_i_ for the CAFB sample at 5.1 K/min amounts to 12.98%, while at 32.8 K/min, it amounts to as much as 32.50%. As expected, increasing the heating rate led to a shift in T_p_ towards higher temperatures (Table 2). This is due to the shorter retention time at higher heating rates, which limits the time-consuming molecular motions taking place before degradation. In addition, the burnout temperature (T_b_) shows an increasing trend as the heating rate is elevated. The rise in T_b_ value from 5.1 K/min to 10.5 K/min amounts to ΔT_b_ = 15.33 °C, while from 21.5 K/min to 32.8 K/min, this rise in T_b_ amounts to as much as ΔT_b_ = 47.86 °C. This clearly suggests that in the case of the CAFB sample, the slow heating rate leads to inadequate combustion. Namely, this can be confirmed based on the received final sample masses (the residual mass) (Δm_(residual)_ in Figure 5a), where it can be seen that at β = 5.1 K/min, the residue inevitably exists due to a series of reasons, such as inadequate combustion and non-combustible components, as mentioned earlier. With the increase in the heating rate, the Δm_(residual)_ was reduced, confirming the above-mentioned statements. This indicates that the CAFB sample is not completely burned out at the lower heating rate, which consumes energy to a certain degree. Considering the R_p_ values (Table 2), there is a decrease in the maximum combustion rate with an increase in the heating rate. It can be noted that the maximum combustion rate is more sensitive to the heating rate when it is expressed by %·min^−1^, than when expressed as %·°C^−1^. This shows that the combustion of the CAFB sample is not only temperature-dependent but also related to the rate of combustion. Within the reaction zone “2” (Figure 5b), the increase in the heating rate causes a decrease in the intensity of the DTG peaks where the width of the DTG peak increases. Consequently, the heating rate has a great influence on the maximum combustion rate, and the observed process is strongly dependent on the operating conditions, considering the oxidative behavior of the CAFB sample. If the particle size of the CAFB sample is small, the high heating rates can lead the maximum combustion rate to decrease (Table 2). Additionally, the combustion reactivity regions are proportional to the height of the DTG peak; therefore, by the observed DTG curves, the lower heating rates produce a more reactive combustion. The reactivity is due to the combustion of volatiles, and the energy released is mainly due to the combustion of the fixed carbon, as can be induced from the last group of the DTG peaks in Figure 5b for the CAFB sample, which was examined. In the case of the smaller particles, the application of high heating rates leads to the single DTG-peak that was observed, but it expands drastically and irregularly (Figure 5b), indicating that the heat released was quickly spent. In that case, the tested material reaches high temperatures for a longer time, and thermal degradation starts later than at lower heating rates. It should be noted that the increase in temperature T_i_ (Table 2) is independent from the particle size consideration, suggesting that temperature gradients inside the tested sample push the initiation of the degradation process to the higher temperatures [49]. This can be a result of the decrease in the heat transfer efficiency [49] for the investigated sample. Nevertheless, it can be observed that the combustion rate for the last group of DTG peaks also decreases with an increase in the heating rate, affecting the heat-released change. Given that increasing the heating rate leads to decreasing the residue at the end of the thermo-analytical experiments, this shows that there is no consistent trend as far as the heating rate is concerned [50]. In summary, it is undeniable that there is influence from both the particle size of the sample used for experiments and the heating rate on the combustion characteristics.

The results presented in Table 2 show that the D_i_ index is correlated with T_i_. The higher ignition index was observed when a better ignition performance was obtained. Index D_i_ was estimated to evaluate the difficulty and speed of the CAFB ignition. Therefore, it can be seen that the CAFB sample is easiest to achieve ignition and shows a more intense reaction in the initial pyrolysis stage at the highest value of the heating rate (32.8 K/min) (Table 2). The lowest value of index D_i_ is identified for β = 5.1 K/min. Index D_i_ significantly increased when the heating rate exceeded 21.5 K/min. Therefore, the faster rate of heating allows a large amount of heat to be supplied to the combustion system in the initial stages so that the increase in the heating rate has a positive response to the ignition behavior.

D_b_ is an important index for characterizing the combustibility of combustibles in the CAFB sample. Since CAFB shows the lowest residue value at β = 32.8 K/min (Figure 5a) for that heating rate, the value of D_b_ is highest (Table 2), and this means that at the considered β, CAFB exhibits the superior burnout performance compared to other heating rates. Namely, the presence of metal(s) impurities in the raw sample can improve the burnout performance of CAFB. Metals and minerals may affect burnout performance by acting as oxygen carriers that promote oxygen transfer to the inside of the CAFB sample. Consequently, a higher residual ash content is an important factor leading to a low D_b_ index (for 5.1 K/min in Figure 5a and Table 2).

Another parameter, flammability index (C), also reflects the reaction intensity in the early stage of combustion, and it is positively correlated with burning stability. The greater the C value, the better the flammability of the CAFB sample, and the more stable the flame of its combustion. Considering four different heating rates, the CAFB sample exhibits a diverse ignition stability. Namely, the highest value of C was achieved at β = 5.1 K/min, while the smallest value of C is present at β = 32.8 K/min (Table 2). This indicates that the combustion stability of the CAFB sample is the best at 5.1 K/min, and the worst is realized at 32.8 K/min. It seems that the best effect of the presence of metals (and/or minerals) in enhancing the combustion stability is shown at a low heating rate.

The index S (comprehensive combustion index) reflects the ignition, combustion, and burnout properties of the CAFB sample. This is integrated quantity, reflecting the CAFB ignition and burnout characteristic indices. The higher value of S indicates the better combustion characteristics of the CAFB sample. It can be seen from Table 2 that the best comprehensive combustion characteristics of the CAFB sample are achieved at the lowest heating rate (5.1 K/min). This is an excellent positive correlation with the previous C index.

Index H_f_ (the intensity index) describes the rate and the intensity of the combustion process. The smaller the H_f_ value, the better the combustion performance. From the results presented in Table 2, we can see that the best combustion performance of the tested CAFB sample was achieved at a heating rate of β = 5.1 K/min.

It can be observed that there is some inconsistency between indices C, S, and H_f_ and indices D_i_ and D_b_. One of the reasons for this distinguishing lies in the relationship (positive/negative), for example, between indexes S and H_f_, and the composition of the studied polymer material. The latter includes information about volatile content (VC) (db—dry basis), the content of ash (db—dry basis), and fixed carbon (FC) (db—dry basis). Therefore, the high ash content may inhibit the combustion of the material, resulting in a decline in combustion performance. It was shown [51] that VC plays an important role in the combustion process; that is, the rapid ignition can increase particle temperature and ignite the FC in advance. Considering that the above information is unavailable for the system investigated in this paper, these are the author*’*s opinions supported by the already published phenomena identified here.

It should be emphasized that there are several factors that affect the kinetic results, such as the material type (the physical and chemical composition of the sample), reaction conditions (heating rate and atmosphere), and experimental data analysis methodology post-processing. Since the kinetic parameters estimated from the oxidative environment may differ significantly from the experiments in the absence of oxygen, it is crucial to study the validity of the kinetic models when the kinetic parameters from the lower heating rate curves are extrapolated to the higher heating rate curves. The latter is important when the obtained kinetic data at lower β’s can be used and applied in the industrial systems where the higher heating rates are present. Therefore, many factors could affect the kinetic parameters, including the process conditions, systematic errors, heterogeneity of the sample, heat and mass transfer limitations, and processing of the thermo-analytical data. Therefore, in this work, a systematic kinetic analysis of the combustion process was approached with the aim of obtaining a realistic and detailed reaction mechanism chart and clarifying the physical and chemical phenomena that occur during the CAFB conversion.

### 3.3. The Kinetic Investigation of CAFB Combustion Process

The objective of kinetic analysis, when applied to thermally stimulated processes, is to establish mathematical relationships between the temperature, degree of conversion, and the process rate. High-quality data are a basic requirement for kinetic analysis. Ensuring reproducibility by carrying out numerous tests under the same measurement conditions is a crucial need for measurement quality. Isoconversional methods are primarily used to analyze the experimental data obtained from processes that involve temperature or time variation (Section 2.4.1). These methods allow us to extract kinetic information from the experimental data and gain a deeper understanding of reaction mechanisms. Therefore, in this part of the study, the analysis of the degree of variation of kinetic parameters (E_a_ and logA) with conversion (α) in the case of the combustion process of the CAFB sample was carried out without a predefined kinetic model, f(α) (Appendix A—Table A1). After this, the model-based (model-fitting) kinetic approach (Section 2.4.2) was applied in order to determine a reliable combination of kinetic models (the reaction mechanism scheme) for the complex combustion process under investigation. The reliability and accuracy of the proposed methods and obtained results were checked through the evaluation of the statistical analysis data.

#### 3.3.1. Isoconversional Kinetic Results from the CAFB Combustion Process

**Figure 6:**
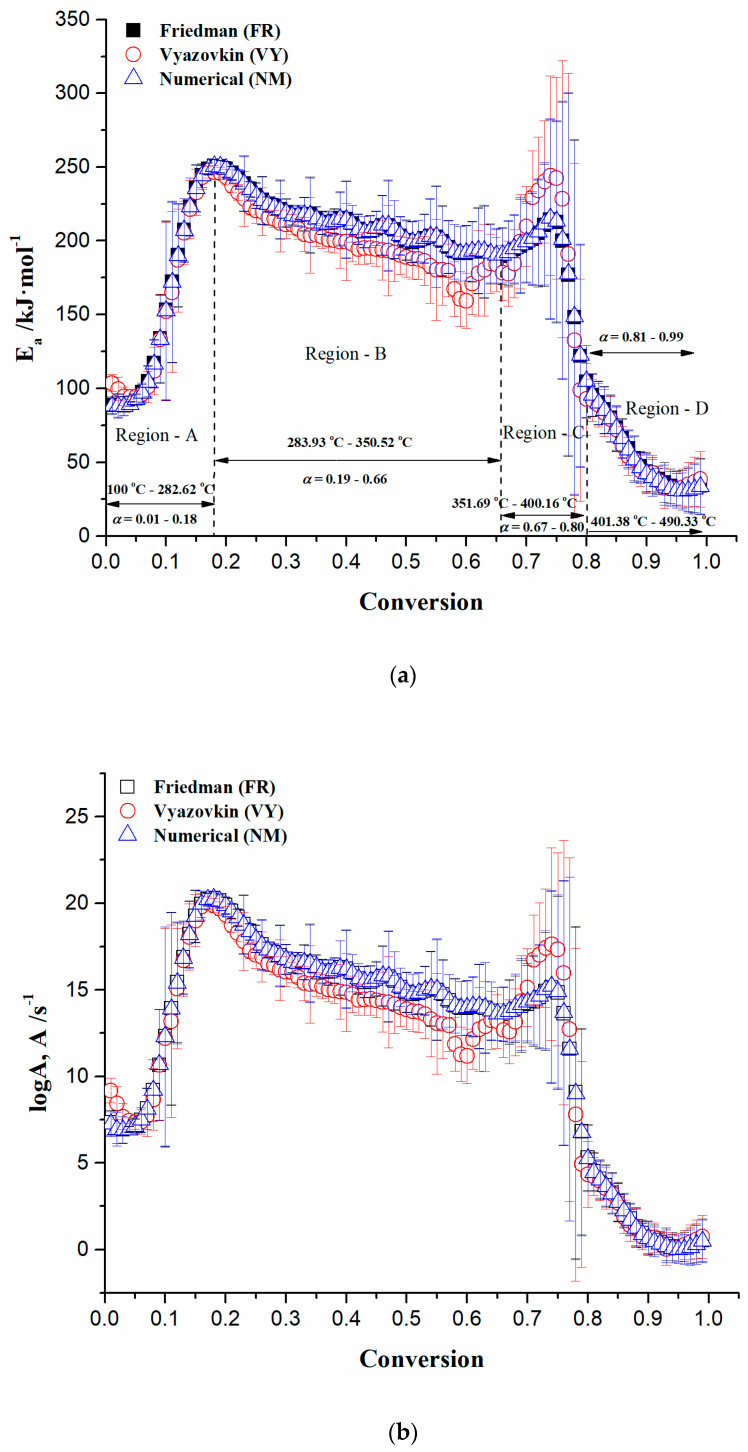
(**a**) E_a_ vs. conversion and (**b**) logA vs. conversion plots, obtained by the application of Friedman (FR), Vyazovkin (VY), and numerical optimization (NM) methods for non-isothermal combustion process of CAFB sample. Characteristic process regions were marked as: “Region A”, “Region B”, “Region C”, and “Region D”, respectively (the conversion and temperature intervals attached to characteristic regions of reactivity are also indicated). Errors (standard deviations) in kinetic parameters are entered as vertical error bars.

Figure 6a,b shows the conversion dependencies on activation energy (E_a_) and logarithm of the pre-exponential factor (logA) values for the combustion process of the CAFB sample under non-isothermal conditions. The observed E_a_(α) and logA(α) plots were obtained by application of Friedman (FR), Vyazovkin (VY), and numerical optimization (NM) methods, which are described in Section 2.4.1. From the obtained isoconversional kinetic results, it can be seen that both E_a_ and logA values exhibit a complex multi-step combustion mechanism with several distinctive regions of reactivity (regions A, B, C, and D, respectively), including various conversion and temperature intervals. All the applied methods (FR, VY, and NM) give very similar trends in the variation of kinetic parameters with the change in conversion (Figure 6a,b). In the isoconversional kinetic analysis, the stage with the elimination of moisture from the sample is omitted, and therefore, the results shown in Figure 6 do not include the removal of water.

From the obtained results, the following general mechanistic conclusions can be drawn (we observe the results obtained through the Friedman (FR) method because a very similar situation is present with other methods):

Region A (Figure 6a: this process region includes lower conversions (α = 1% − 18%) and temperatures between 100 °C and 282.62 °C)—In this part of the process, there is an increase in the activation energy value from 88.48 kJ·mol^−1^ to 250.81 kJ·mol^−1^. The observed reactivity zone of CAFB is associated with reactions related to triacetin (TA) (C_9_H_14_O_6_) (glycerol triacetate) thermo-chemical conversion. This stage may include the metal-assisted hydrolysis of TA (with presence of H^+^), impacted by the previous reaction stage (the hydrolysis can be initiated through the contact of the TA molecule with water vapor in the presence of metal(s) incorporated inside the filters), occurring in a gas phase [52,53]. The activation energy values can be in the range of 2 kJ·mol^−1^–150 kJ·mol^−1^ [54,55]. As the products of the hydrolysis step, the glycerol and acetic acid are formed. Glycerol is regarded as a renewable and industrially important source of the raw material for the production of value-added chemicals, such as glycerol carbonates, esters (acetins), and ethylene glycol, among others [56,57]. However, within this process stage, the glycerol combustion reaction pathway takes place, considering its higher auto-ignition temperature [58]. The glycerol oxidative degradation in the presence of oxygen may occur through the cascade of reactions at elevated temperatures, where the main source can be just cigarette filters. The reactions may proceed to a termination stage, ultimately resulting in the toxic carbonyl-containing products. However, observing our case, the termination phase can be concluded according to the values of the activation energies (E_a_) obtained in the later part of Region A. Namely, the presence of oxygen clearly enables chemical transitions that are not possible in an inert atmosphere, referring to our previously published work [12]. In line with this, the thermo-chemical conversion study may confirm an ability for oxygen to unlock an entirely new network of chemical conversions, which proceed through a low-temperature reaction pathway. Therefore, we can assume with a high degree of probability that oxygen can be inserted into a glycerol molecule and generate radical species via H-abstraction. In the presence of water traces/metal species [M^4+^—such as TiO_2_] and the oxidizing agent, the glycerol undergoes oxidative degradation via hydrogen abstraction. This allows the opening a lower C‒H bond cleavage activation energy pathway, producing as the main product 1,3-dihydroxyacetone (DHA) (IUPAC: 1,3-dihydroxypropan-2-one), with an activation energy of 195 kJ·mol^−1^ [59] (at α~0.12, there is E_a_ = 190.34 kJ·mol^−1^ (Figure 6a)) (^●^OH radical species, which can be formed from water oxidation, may further lower the energy barrier for obtaining the product of this reaction). It should be noted that in addition to DHA, the production of glyceraldehyde (GLYAD) can be achieved since it requires a slightly lower activation energy of 183 kJ·mol^−1^ [59] (see Figure 6a).

Region B (Figure 6a: the observed process region is the widest and includes the conversion range of α = 19% − 66% and temperatures between 283.93 °C and 350.52 °C)—The actual process region belongs to the thermo-chemical conversion reactions of cellulose diacetate (CDA) in the CAFB sample (Figure 4). The conversion of CDA starts with the deacetylation process through thermally induced hydrolysis, where the CAFB fiber morphology can be changed [60]. Since the activation energy for the deacetylation of CA-fibers amounts to approximately 43.10 kJ·mol^−1^ [61], we can assume that the deacetylation process had already occurred much earlier, before entering Region B. It should be noted that the obtained mean value of the activation energy relating to Region B (E_a(mean-B)_ = 211.62 kJ·mol^−1^) corresponds to the activation energy for CA with a higher degree of substitution (DS (see results in Section 3.2)) [62]. Since the cellulose belongs to relatively stable polymers with respect to thermal oxidation, when compared, for example, with polyolefins, the mechanism of its degradation is much more complex. The kinetics of the thermo-oxidative process are affected by the temperature and the oxygen accumulation. The oxidation of cellulose by oxygen can start at different sites of 1,4-β glucopyranosyl monomer units of the cellulose chain. As proposed by Shafizadeh and Bradbury [63], the preferred sites of the primary oxygen attack are carbon atoms in positions 1 and 4 of the glucopyranosyl units, but an attack focused on carbon atoms 2 and 3 linked with alcoholic groups and a carbon atom at position 5 linked with the methylhydroxyl group cannot be excluded either. Namely, the combustion of cellulose may consist of a series of complex chain reactions [64]. In that context, the cellulose glycosidic bond cleavage represents a primary reactive center for the further development of the reaction mechanism. As can be seen from Figure 6a, the activation energy value inside Region B gradually decreases from E_a_ ~ 250.44 kJ·mol^−1^ up to E_a_ ~ 189.38 kJ·mol^−1^ (at α = 66%). However, the obtained values of E_a_ are higher than the range of activation energies estimated for the degradation of cellulose from lignocelluloses materials (171.04 kJ·mol^−1^ − 179.54 kJ·mol^−1^) [65], but they are within the range of E_a_ values for degradation of the pure cellulose (100 kJ·mol^−1^ − 250 kJ·mol^−1^) [66].

Due to the presence of liberated water vapor from the previous reaction stages and the existence of a water-tolerant solid acid catalyst (such as TiO_2_) in the CAFB sample, the glycosidic bonds can be easier protonated and hydrolyzed to the glucose. The acid catalyst can significantly promote a scission of the glycosidic (C‒O‒C) bonds in the cellulose to yield glucose through hydrolysis. In addition, with the breaking of glycosidic bonds in combination with oxidation (O_2_), cellulose can be transformed into gluconic acid with a high selectivity due to its relatively higher stability than glucose. Therefore, we can assume that there is a selective activation of the C‒O bonds in cellulose of the CAFB sample during its combustion process, where catalytic scission is taking place, with an activation energy of 219 kJ·mol^−1^ [67,68]. This is quite comparable to the values of the activation energy in Region B. However, since it is a complex reaction mechanism that can proceed through several elementary steps, their number and order cannot be determined by applying the isoconversional kinetic analysis. This is supported by the fact that the transglycosylation mechanism can be included [69], occurring in noncatalytic manner, with the lower activation energy of approximately 209 kJ mol^−1^ (this value also belongs to the E_a_ values present in Region B at α ≈ 0.45 (Figure 6a)), which is in good agreement for α-cyclodextrin conversion at the higher temperatures [67]. The answer to these questions (in the form of “confirm or it is opposite”) can be given by model-based kinetic analysis, which communicates information about the contribution of the individual reaction steps to the entire process and gives us reliable kinetic parameter magnitude domains within an individual reaction channel.

In addition, the first, sharp exotherm peak at DTA curve (Figure 5d) is located inside the Region B, so, the flaming combustion of the volatile compounds takes place in it, which were produced during the rapid mass loss of the CAFB sample.

Region C (Figure 6a: this process region occurs in the conversion range of α = 67% − 80% and in the temperature interval between 351.69 °C and 400.16 °C)—This stage of the process is characterized by an increase in the activation energy (E_a_) value from 191.05 kJ mol^−1^ to 214.48 kJ mol^−1^ (from α ~ 67% to α ~ 74%) and then reducing to the value of 104.37 kJ mol^−1^ (α ~ 80%). The considered region belongs to the char-forming processes [70] in the thermal degradation of cellulose during the CAFB conversion at the expense of volatilization. This process occurs at the transition between “III” and “IV” zones in Figure 5c (it is characterized by an appearance of the inclination angle on the TG curve within region “3” in Figure 5a). It can be observed that the obtained E_a_ values are quite high for this part of the process, which may indicate that the heat produced by the char-forming reactions is not removed from the system efficiently. This can cause the temperature of the reacting system to increase, thus inducing the higher activation energy pathway. However, if the accumulated water content is high, then it can enhance the char formation. However, it should be noted that char usually forms through the secondary reactions of the depolymerization product, but distinct char types may exist (the primary char, which retains a similar gross structure to a substrate, and the secondary char, which is fluffy and unstructured) [71]. Since the actual distribution of E_a_ values is estimated within Region C and the resulting mean value of E_a(mean-C)_ = 183.90 kJ mol^−1^, this indicates that the thermal transport barrier effect can be important. Namely, the surface char tends to insulate un-burnt material from the heat generated in the gas-phase combustion. Therefore, a delicate balance must exist between the amount of char and the degree of fire resistance, because the temperature, and consequently, the rate of thermal degradation, can increase enormously as exothermal (bond-forming) charring reactions take place. This would be the introduction to the next stage of the process.

Region D (Figure 6a: this process region occurs in the conversion range of α = 81% − 99% and in the temperature interval between 401.38 °C and 490.33 °C)—In this region, there is a progressive decrease in the value of the activation energy (E_a_) from 95.22 kJ mol^−1^ (for α ~ 81%) until the very end, with 31.71 kJ mol^−1^ (α ~ 96%). The occurrence of an exothermic event characterized by a broader DTA peak at 435.89 °C (Figure 5d) was located inside Region D. This belongs to the oxidation reactions occurring in the charred sample (the combustion of the solid) [64]. The latter is characterized by the second part of the TG curve (after the inclined portion) within region “3” in Figure 5a. Region “4” (Figure 5a) represents un-burnt residue, regulated by the heating rate intensification, and it is attributed to a very slight increase in E_a_ at the very end of the complete CAFB conversion process (Figure 6a).

Using the procedure described in Section 2.4.1., logA—conversion dependency was determined by the application of the Friedman (FR), Vyazovkin (VY), and numerical optimization (NM) methods, respectively. The logA(α) profiles for the CAFB combustion process are shown in Figure 6b. It can be seen that the estimated logA values follow the trends of E_a_’s shown in Figure 6a, whereby the magnitudes of pre-exponential factors are in complete agreement with the range of the calculated E_a_ values by means of model-free methods for the combustion process of interest.

Based on the obtained kinetic data from the isoconversional (model-free) kinetic methods, the conversion fit (through the fitting procedure of TG signals) between experimental and calculated results was performed. For the optimization and curve fitting, the Kinetics Neo software (Product version: 2.7.0.11, Build date: 29 January 2024) uses the non-linear least square approach. In order to achieve the best coefficient of determination (R^2^) in the calculation fit to the experimental data, the kinetic parameters were optimized.

**Figure 7:**
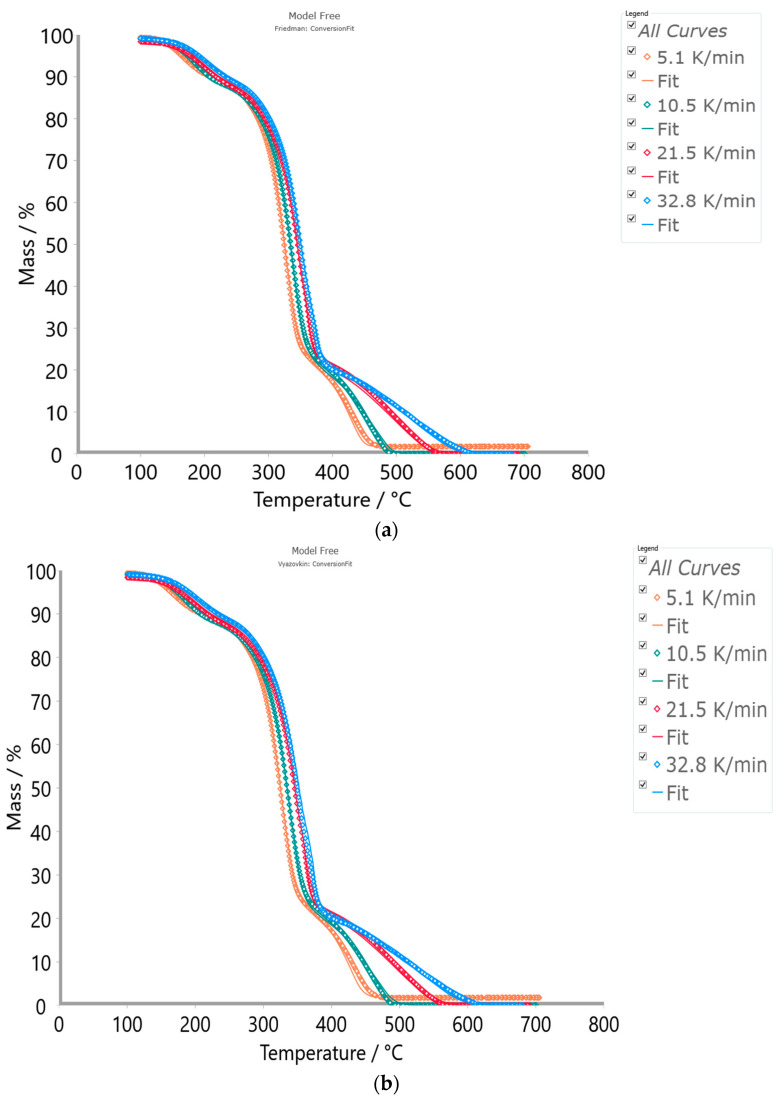

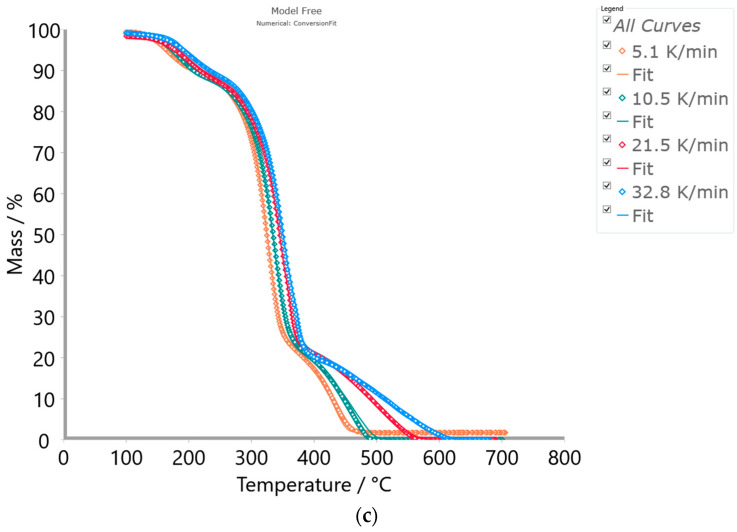
The “conversion-dependent” model-free data fit to the experimental TG signals at different heating rates (5.1, 10.5, 21.5, and 32.8 K/min) for the (**a**) Friedman (FR), (**b**) Vyazovkin (VY), and (**c**) numerical optimization (NM) methods, in the case of complex (multi-step) combustion process of CAFB sample (the corresponding values of adjusted R-Square (R^2^) are as follows: FR − R^2^ = 0.99990; VY − R^2^ = 0.99987; NM − R^2^ = 0.99991).

Figure 7a–c show the conversion fit between experimental TG curves and TG curves constructed from the optimized kinetic parameters estimated by the Friedman (FR), Vyazovkin (VY), and numerical optimization methods for the combustion process of the CAFB sample.

As can be seen from Figure 7, all three methods show very high quality in the fit to the experimental thermo-analytical data in the entire range of the observed temperatures at given heating rates. Based on the obtained results, we can see that these methods perform the kinetics complexity of the investigated process very well, confirming its multiple-step reaction nature. The model-free methods are suitable for dynamic measurements, where at least three heating rates are required, and they are suitable for the data evaluation of each reaction point. The resulting isoconversional kinetic parameters hold all the information about the oxidative degradation of the CAFB sample, and they can replace standard Arrhenius kinetic constants with the formation of the standard kinetic equation and, hereby, the assumption of the first-order for the f(α) function (Figure 6b). Based on the obtained quality of fitting, the methods have the following order: NM > FR > VY. Given that NM and FR methods exhibit very similar R^2^-values, we decided to use the FR model-free approach as the zero approximation for solving the CAFB degradation mechanism scheme by the model-based (model-fitting) kinetic method (see later).

Prior to the application of the model-based analysis, the preliminary determination of the reaction model types involved in the degradation mechanism based on the FR isoconversional plots was explained (see the text below).

##### Preliminary Determination of Reaction Model Types for CAFB Combustion Process

**Figure 8:**
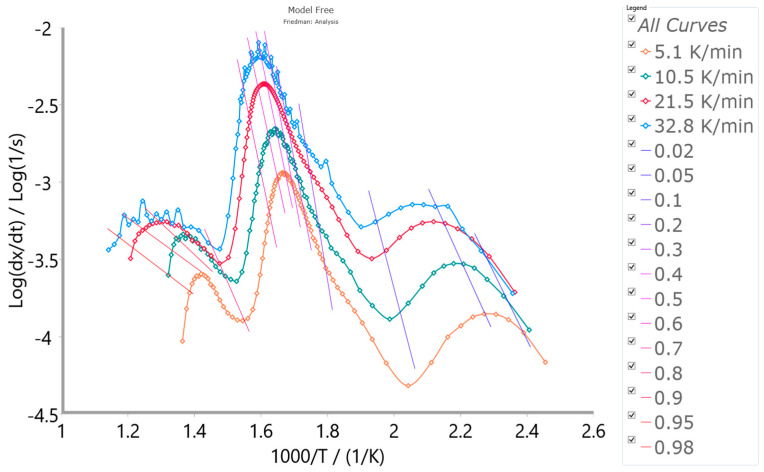
Friedman (FR) isoconversional plots at different heating rates (conversion (α ≡ x) goes from right to left) for the non-isothermal combustion process of CAFB sample.

Based on the performed model-free (isoconversional) analysis, the appropriate Friedman (FR) isoconversional plots (at selected conversion values (α ≡ x)) were created, and these plots are presented in Figure 8.

From the Friedman (FR) analysis presented in Figure 8, on the basis of the isoconversional line inclination angle to ordinate axis, the arguable reaction type can be decrypted. Based on the forms of the FR isoconversional plots, it is obvious that the investigated CAFB combustion represents a multi-step reaction process. Three peaks are discernible at the considered heating rates: the first one is positioned at lower conversions, while the second and third ones are positioned at the medium and the high conversions. From the isoconversional lines (shades of “blue” lines to the particular conversion value—lower conversion zone), the peak slope is much steeper than isoconversional lines, which suggests the presence of an accelerated reaction (the process is accelerating) [72]. The presence of an accelerated reaction can be detected based on the fact that the slope of isoconversional lines is gentler than the first peak slope on the FR plots. Additionally, linked with the second peak, the isoconversional lines (shades of “pink” lines to the particular conversion value—the medium conversion zone) are slowly taking on a parallel format, exhibiting a little lower slope in the experimental results. This may suggest the presence of a reaction with deaccelerating character (such as contracting geometry models or n-th order reaction models) [72]. In the latter phase, with high conversion values (third peak), the isoconversional lines (shades of “red” lines to the particular conversion value) abruptly change slopes compared to the second peak in Figure 8. In the considered case, the distances between constant conversion points plots are increasing with the increase in heating rate from 5.1 K/min to 21.5 K/min (except for 32.8 K/min), which suggests accelerating, i.e., the presence of the accelerating reaction [72]. For the indifferent processes (for example, such as the first order/second order reactions), the distances between the constant conversion points should be more or less the same (this would roughly correspond to the case at the second peak in Figure 8). Therefore, once the kinetic model type has been properly selected, one can proceed to the non-linear regression to optimize the model parameters into the one integral scheme of the reaction mechanism of the process being examined. However, the main drawback of isoconversional methods is the inability to recognize the parallel and independent reactions. Therefore, the above-described methods can represent multiple-step reactions without parallel reaction steps, although there is no detailed knowledge about the reactions. If parallel and independent reactions take place, only the mean values of E_a_ are considered. On the other hand, the model-based method may provide the complete kinetics information (enclosed kinetic triplets), which can be verified by the best kinetic models, describing the process in the real physicochemical bases.

#### 3.3.2. Results Obtained from Model-Based Kinetic Analysis of the CAFB Combustion Process

Within the model-based computation machinery, over multiple rounds of the multivariate non-linear regression (MVNLR) optimizations, the reaction scheme that best describes the entire combustion process of CAFB was established. The obtained models were coded as the p:, Model. This mechanistic scheme includes two parallel consecutive reaction steps and one independent single-step reaction, presented through Equations (15)–(17) (note: the reaction mechanism scheme does not include the stage with moisture evaporation—omitted from this analysis):

(15) $$A\;\overset{Fn}{\to }\;B\;\overset{Cn}{\to }\;C$$

(16) $$D\;\overset{F2}{\to }\;E\;\overset{A3}{\to }\;F$$

(17) $$G\;\overset{Nk}{\to }\;H$$

where A, D, and G represent reactants; B and E are intermediate species; while C, F, and H represent the products. The first sequential stage includes step A→B, described by the n-th order chemical reaction (Fn) (Appendix A—Table A1), while step B→C within the same stage was described by the n-th order reaction with autocatalysis (Cn). The second sequential stage contains step D→E, described by the second order chemical reaction (F2), and step E→F, described by the three-dimensional (3-D) growth of the nuclei (Avrami equation) model (A3). The single-step reaction stage characterized with G→H (Equation (17)) was described by the Nakamura crystallization model (Appendix A—Table A1) (Nk), which includes the Avrami nucleation model via Arrhenius behavior and the Lauritzen–Hoffman (L-H) nucleation theory grounded on the non-Arrhenius behavior. The reader can see more details about this model in our previously published paper [12].

Table 3 lists the characteristics of the established kinetic models for description of the CAFB combustion process, including appropriate concentration equations, the rate-law equations for elementary steps, required kinetic parameters, and other kinetic/geometrical exponents, considering all the chemical species involved in thermo-chemical conversion.

It should be noted that the value of E_a_ considered in the case of the isoconversional analysis (see above) should be called the “apparent activation energy value” and could differ from the true activation energy value in the chemical sense. The activation energy E (Table 3) can be called true because it is a part of the complete kinetic information of the system—the finally formed kinetic triplet [A, E, f(α)—best optimized for the process of interest]. The function f(α) represents the reaction type, and it was also called the mechanism function. It represents the dependency of the reaction rate on the conversion and can be treated as a mathematical description of the reaction mechanism (Table 3).

The corresponding mass balance Equation is expressed as

(18) $$\begin{array}{l} Mass=Initial\;mass\\ \;-\;Total\;mass\;change\\ \;\times \;[ctb.\;\left(a\to b\right)\\ \;\times \;\int \left[\frac{d\left(a\to b\right)}{dt}\right]dt\\ \;+\;ctb.\;\left(b\to c\right)\\ \;\times \;\int \left[\frac{d\left(b\to c\right)}{dt}\right]dt\\ \;+\;ctb.\;\left(d\to e\right)\\ \;\times \;\int \left[\frac{d\left(d\to e\right)}{dt}\right]dt+ctb.\;\left(e\to f\right)\times \int \left[\frac{d\left(e\to f\right)}{dt}\right]dt+ctb.\;\left(g\to h\right)\times \int \left[\frac{d\left(g\to h\right)}{dt}\right]dt] \end{array}$$

where ctb. represents the contribution of a given elementary reaction step.

The values of the kinetic parameters (/kinetic exponents) and reaction type functions in each kinetic triplet for the individual reaction steps and their relevant contributions, together with the corresponding temperature intervals in which these steps are taking place, are listed in Table 4.

Since the apparent activation energies and pre-exponential factor significantly vary with conversion by application of isoconversional methods (where FR and NM are the most accurate methods) (Figure 6 and Figure 7), there are α-regions where standard deviations (errors) have larger magnitudes, introducing the noise data that cannot be ignored. In this regard, the kinetic parameters obtained in these conversion portions would be unreliable, but they should not be discounted completely. If there is a change, is it gradual or can it be related to some feature in the data? Solving this problem allows for application of the powerful cutting-edge mathematical calculations incorporated within the Kinetics Neo software using the MVNLR procedure, enabling the creation of the best kinetic reaction mechanism, i.e., different kinetic models (Table 4), and they can be compared statistically (see later). Therefore, this approach has none of the disadvantages that can be observed when using model-free methods. The model-based kinetic analysis offers the possibility of visual design for kinetic models with an unlimited number of reaction steps connecting in any combination. Therefore, the results presented in Table 4 are the best optimized kinetic parameters for the investigated process. The next task is an interpretation of the results listed in Table 4; therefore, their adequate analysis is needed, giving them a physical meaning. In the following lines, an explanation of the complete mechanistic scheme of the CAFB combustion process is provided.

(a) Step G→H (Nakamura (Nk) crystallization model) (Equation (17)) is occurring in the temperature range of ΔT = 120 °C − 260 °C ‒ This model can be presented by the general rate-law Equation, expressed through the extent of reaction (α), as [12]

(19) $$\frac{d\alpha }{dt}=A\cdot f\left(\alpha \right)\cdot K\left(T\right)=A\cdot n\cdot \left(1-\alpha \right){\left[-ln\left(1-\alpha \right)\right]}^{\frac{\left(n-1\right)}{n}}\cdot exp\left[-\frac{U^{*}}{R\left(T-T_{\infty }\right)}\right]\cdot exp\left(-\frac{K_{G}}{T\cdot \Delta T\cdot f}\right),$$

(K(T) is the temperature-dependent growth rate) where the model is approximately defined for the temperature range between T_∞_ and T_m_ (T_∞_ is the hypothetical temperature, where all motion associated with viscous flow ceases, normally chosen as T_g_ − 30 K) (∆T is the degree of supercooling, and f represents the correction factor, which reflects the reduction in the latent enthalpy of fusion). It is interesting to note here that this transformation is completely identical (up to ~260 °C) to the one that occurs in the case of the CA fiber pyrolysis process but with slightly different parameter values within the Nk model [12]. The actual process occurs in the temperature range that includes the physical event between the glass and melting temperatures, with plasticizer vaporization into the gas-phase from the polymer matrix, as well as the deacetylation reaction (Section 3.3.1). In the current case, the established kinetic parameters from the model-free kinetic approach related to this T-range (Figure 6) must be taken strictly as the apparent (effective) parameters, because it is limited in the small temperature range, just below T_m_ temperature (as if this part of the process were considered under isothermal conditions). Therefore, the Nk parameters listed in Table 4 can be considered as those who have their own effective values, independent from those in the isoconversional analysis [12]. The observed phenomenon is related to the miscibility of the system (plasticizer + cellulose derivatives physical behavior), and details related to this issue can be found in our previous work [12]. Considering the already described properties of the examined system during its thermo-chemical conversion, the amount of plasticizer has an important role in the extent of the plasticizer expulsion, from a localized CA-rich domain [12]. Given the higher DS value, we can assume that the present amount of plasticizer improves segmental mobility substantially, making the crystal perfection and the formation of new crystals easier, favoring the crystallization process. Consequently, the development of crystallinity in the thermally treated cellulose derivative induced by a plasticizer can be expected [12]. Therefore, the reaction step G→H described by Equation (19) (see also Table 3) can be attributed to the isophase transitions of cellulose, occurring below the thermal degradation temperature [73] (pp. 1–100). These transitions depend on the estimated glass transition temperature value(s). However, experimental T_g_ was not identified in this study, whereby T_g_ value in Table 4 (T_g_ = 120 °C) represents the theoretically determined glass transition temperature for the examined system. The obtained T_g_ value is in the range of T_g_ for the plasticized cellulose derivative sample [74] (pp. 21–23). It should be noted that the T_g_ calculated in this paper differs (the lower value) from the T_g_ value obtained in the case of pyrolysis of cigarette butt fibers composed by CTA (cellulose triacetate) (=182.68 °C − experimentally determined) [12]. Namely, an increase in the amount of plasticizer can drastically lower the glass transition temperature. The detection of isophase transition can be performed through the ratio T_g_/T_m_ = 0.66 [75,76] (pp. 129–189), linked to cellulose macromolecule. For our study, this ratio amounts to T_g_/T_m_ = 0.50 (with deviation of 0.16), and it is a characteristic value for a cellulose derivative. However, the certain difference that occurs here, in relation to the obtained T_g_/T_m_ ratio under pyrolytic conditions, lies in the presence of cellulose derivative types in the investigated sample, i.e., cellulose triacetate (CTA) [12] and the cellulose diacetate (CDA) (in this work). The main difference between triacetate and diacetate lies in the degree of acetylation, which leads to differences in their properties and applications. The obtained value of T_g_ (Table 4) is located far below the range of the T_g_ values identified for the pure cellulose (217–227 °C (for cellulose, this refers to primary α_1_ glass transition)) [77,78], where this represents an indication of the strong plasticization effect of TA, lowering the T_g_ [12]. With a significant increase in the plasticizer amount, the probability of the occurrence of α transition (α-relaxation) is very high, moving it towards lower temperatures [12]. Therefore, the step G→H can be attributed to the glass-to-rubber transition of the CAFB sample, with the presence of α transition, which is also described in ref. [12], in the case of the pyrolysis process.

Considering the value of Avrami’s dimension parameter ((n) = 0.374) (Table 4), it suggests the one-dimensional growth of nuclei, controlled by diffusion (primary crystallization). This may occur in the early stage of the process where the influential presence of the plasticizer can accelerate the dynamics of CDA crystallization, in a similar manner as described in ref. [12]. The nucleation is influenced by the presence of the plasticizer, acting as a “diluent” for the CDA/TA mixture. The nucleation represents an initial step in this process in which crystalline structures begin to form within the polymer matrix. It is a crucial step in the crystallization process, because it determines the number and size of crystalline domains that will form. When the nucleation is the rate-determining step, the activation energy is expected to be a negative (K_G_ value in Table 4). A very similar situation was identified under the pyrolysis process conditions [12]. The negative and large value of the nucleation parameter (K_G_) (Table 4) supports the fact that the presence of metal particles reduces the energy needed to create a new crystal surface and then accelerates the crystallization rate.

However, logA has a positive value (=2.411 (Table 4)), giving a positive sign over the entire right-hand side of Equation (19) (see Table 3), which controls the temperature dependence of the nucleation rate, related to structural changes. In that case, the overall crystallization rate increase with temperature; therefore, as the temperature further progresses, the concentration of the “crystallized” product increases (designated as “H”) (Equation (17)). In this case, the opposite phenomenon occurs to that observed in the case of the pyrolysis process [12], where the change in crystallization rate (correlated with a change in the crystallization mechanism) takes place, while here, it does not happen. Namely, in the considered case, we have the acceleration of secondary crystallization as temperature rises and by increasing the melt temperature (=240.695 °C (Table 4)). There is a transformation of cellulose I less-ordered crystallites into the formation of ordered cellulose II crystallites [12] where, however, the spherulitic growth is governed by primary crystallization, which, in turn, affects the extent to which the secondary crystallization can take place. Given the obtained parameters of the Nk model (Table 4), the geometry of the secondary nucleus has not changed (i.e., the crystallites keep their shape in the one-dimension (rod-like crystallites)), whereas secondary crystallization takes place at a smaller scale and does not require major molecular re-arrangement in the manner that primary crystallization does (since it occurs in the already established interlamellar regions); therefore, it is a possibility that it locally progresses at a much higher rate. This may produce a larger cellulose II yield from the secondary phase of the actual process (the smaller spherulites lead to a more compact lamellae closer to the spherulite nucleus and, therefore, less interlamellar space). In addition to the indicated facts, the contribution of secondary crystallization may decrease as the “cooling” rate increases in the non-isothermal crystallization [79], taking into account that double melting temperatures are not registered (Table 4). Comparing these results with those obtained in our previous work [12], important differences are reflected in the rate of crystallization of the new product and in the appearance of an additional reaction step, which is related to the change in the dimensionality power exponent of the crystal growth.

**Figure 9:**
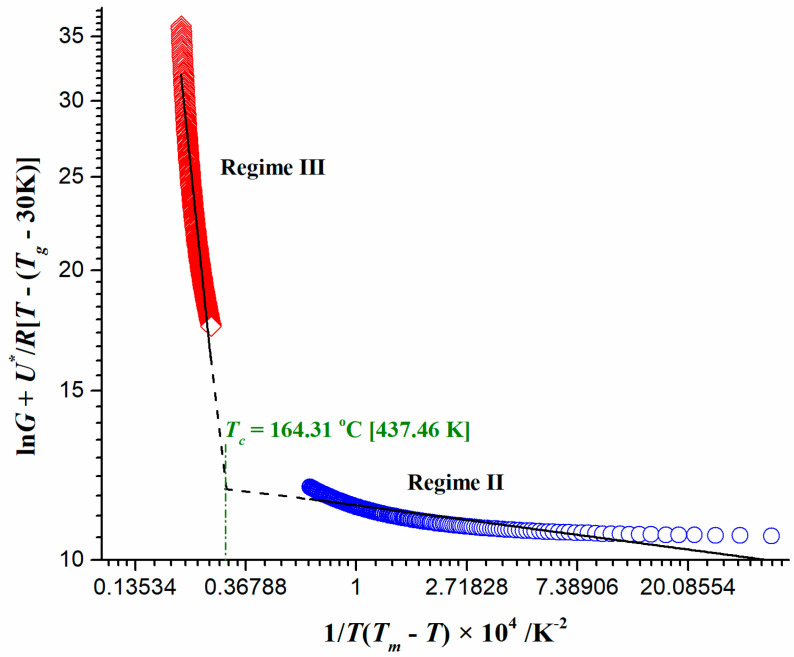
Lauritzen–Hoffman (L-H) plot for T_m_ = 513.845 K, U* = 6300 J·mol^−1^, and T_∞_ = T_g_ – 30 K. (the crystallization regime transition analysis: Regime III—K_G_^III^ = −1.10 × 10^4^ K^2^; Regime II—K_G_^II^ = −0.54 × 10^4^ K^2^); The crystallization temperature amounts to T_c_ = 164.31 °C [T_c_ = 437.46 K].

In order to obtain a more detailed insight into the crystallization mechanism from the melt, we applied the Lauritzen–Hoffman (L-H) secondary nucleation theory (from that theory, the kinetics become the rate at which the polymer grows on the surface, or the lateral growth rate, in comparison with the growth rate onto the polymer extending the chain—the secondary nucleation rate), for establishing the kinds of crystallization mechanisms [80] for our observed polymer system. Figure 9 shows the L-H plot of lnG + U*/R[T − (T_g_ – 30 K)] against 1/T(T_m_ − T) (G—is the crystal growth rate, U* = 6300 J mol^−1^, T_g_ is the glass transition temperature (K), and T_m_ is the melt temperature (K)).

As can be seen from Figure 9, there are two crystallization mechanisms in the transition regimes III-II. The crystal growth rate (G) is mainly controlled by diffusion rate, g, and the secondary nucleation rate, i [81]. When i is much smaller than g, the secondary nucleation rate is much lower than the lateral growth rate. Before new crystal nuclei are formed, the molecular chain has completed the growth process, which belongs to the crystallization mechanism, I (Regime I).

When the secondary nucleation rate i increases and the diffusion rate g decreases but i is slightly greater than g, the new crystal nuclei have been generated before the old lamellas have completed the growth process; that is, multiple crystal nuclei grow at the same time, which belongs to the crystallization mechanism, II (Regime II). Finally, when the nucleation rate i is much greater than the diffusion rate g, before the old crystal nuclei grow, many new crystal nuclei are produced, and this belongs to the crystallization mechanism, III (Regime III). In our considered case, only the transformation of mechanism III to II is observed (Figure 9). Therefore, there are two distinctive regime transition behaviors. The appropriate values of K_G_ can be obtained from the slope of the straight lines shown in Figure 9 (K_G_^III^ = −1.10 × 10^4^ K^2^ and K_G_^II^ = −0.54 × 10^4^ K^2^). The ratio of secondary nucleation constants (K_G_^III^/K_G_^II^) is approximately equal to 2 (=2.04), which is predicted by the secondary nucleation theory (conforms to L-H theory). Therefore, when the crystallization temperature range was ≈ 115~153.44 °C, the crystal morphology was made of banded spherulites, corresponding to mechanism III. When the crystallization temperature range was ≈ 209.12~256 °C, the crystal morphology was made of non-banded spherulites, corresponding to mechanism II. It should be noted that there is a discontinuity detected in Figure 9, but the identified regimes exist in the range of the crystallization temperature explored. The crystallization temperature (T_c_, Figure 9) corresponds to the “inflection point” and represents the temperature at which the crystallization mechanism changes [82].

The presented results reflect the formation and structure of cellulose spherulites during the thermally induced conversion of CAFB in the framework of morphological regulation and supramolecular architecture of polymer spherulites. Therefore, the “broken line“ in Figure 9 represents the morphological transition from “negative” cellulose I spherulites (banded) to “positive” cellulose II spherulites (non-banded).

In our specific case, the way to obtain the crystalline form of cellulose II (G→H step—Equation (17)) (as the product (“H”)) contributes to the overall conversion process of the CAFB sample of 3.7% (Table 4), while in the case of pyrolysis (when CTA is part of the experimental sample) it amounts to 7.3% [12].

(b) The consecutive reaction step D→E→F (Equation (16)) occurs over the entire process temperature range, whereas the first reaction in the series, D→E, takes place in the T-interval of ΔT = 100 °C − 350 °C. This reaction can be attributed to TA (triacetin) hydrolysis in the presence of water vapor (H_2_O) to glycerol and acetic acid [83,84], proceeding as in a form of Equation (20):

(20)
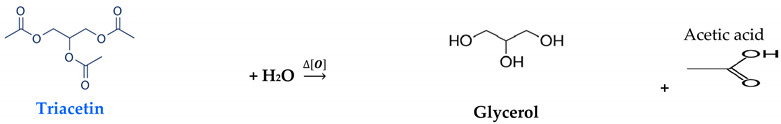

where glycerol (glycerin) represents the intermediate specie produced in the reaction D→E. The current step proceeds through the second-order reaction kinetics (F2) with respect to concentration of TA as the reactant within CAFB. The oxidative medium (oxygen) can act as a mild catalyst, reducing the E value [85] (86.526 kJ mol^−1^, Table 4).

Glycerol, as an intermediate product, represents an emerging renewable bio-derived feedstock that could be used as a source for producing hydrogen through a steam reforming reaction [86]. In addition, the glycerol is the source of energy carriers and chemicals, and it represents the reactant in the next reaction, E→F, in the observed sequential series.

Step E→F occurs at much higher temperatures during thermo-chemical conversion of the CAFB sample, and it is related to glycerol interaction with the metal catalyst surface (for our system, this is TiO_2_ present in the examined specimen), proposed by the three-dimensional Avrami–Erofeev model (A3) (Table 3 and Table 4). This means that the Lewis acid-base interaction of glycerol with the Ti active site of the surface includes the electron transfer from glycerol to the catalyst. Namely, after adsorption of glycerol on the TiO_2_ surface (the nucleation center), it can be converted to acrolein by dehydration. This step is essentially much more complex, where the dehydration of glycerol to acrolein starts with the losing of a water molecule through surface-assisted central hydroxyl elimination. The most energetically favorable arrangement for this transformation involves the lowest energy reaction pathway, where, after the glycerol adsorption on the TiO_2_ surface, the dehydration reaction started with the abstraction of H_2_ by an O_2c_ atom of the surface that led to the chemical adsorption of glycerol on the surface [87]. This reaction requires a minimum activation energy value of 50.626 kJ mol^−1^ [87], which differs from our value only by 16.318 kJ mol^−1^ (E = 66.944 kJ mol^−1^, Table 4). This is a clear indication that the dissociative adsorption of glycerol on the TiO_2_ surface is an easy and fast step (compared to the previous slower step (D→E)). Thereafter, the TiO_2_ surface simultaneously takes H1′ and Oβ atoms (in glycerol structure numbering) [87], while a double bond is concertedly created between Cα and Cβ, producing (growth) of a new product (acrolein) in the spatially schedule (three dimensional, 3-D). In oxidative conditions, within practical terms, the smell of burning fat is caused by the breakdown of glycerol in burning fat into the acrolein. At this point, the listed kinetic parameters for reaction step E→F in Table 4 corresponds to the E_a_ and logA values estimated by isoconversional methods (Figure 6a,b) at the conversion of α ~ 86% within Region—D. This participates in the acrolein smoke component yields as a toxicant, which apparently appears during the combustion of the CAFB sample. On the other hand, for the previous step (D→E), the kinetic parameters presented in Table 4 correspond to the E_a_ and logA values from the isoconversional methods at conversion of α ~ 5% within Region—A (Figure 6a,b). Obviously, there is an excellent agreement between model-free and model-based results. Contributions of reaction steps D→E and E→F (Equation (16)) to the entire combustion process of the CAFB sample are 11% and 14%, respectively (Table 4).

(c) The next consideration relates to the second consecutive reaction step, including A→B→C, where we have an overlap of temperature regions on their occurring, as ΔT_A→B_ = 260 °C − 450 °C and ΔT_B→c_ = 300 °C − 700 °C (Equation (15)). The first reaction in the series (A→B) can be attributed to the cellulose chemistry of transglycosylation, which occurs towards higher temperatures of the CAFB combustion process. The range of activation energies for cellulose transglycosylation covers the values between 199.6 kJ mol^−1^ and 250 kJ mol^−1^ [88,89]. In the case of CAFB thermo-chemical conversion, the current reaction (cellulose activation) takes place via n-th order kinetics (with fractal order value of n~2.547) with activation energy of E = 229.505 kJ mol^−1^ (Table 4). Therefore, transglycosylation is the most likely mechanism for description of the glycosidic bond cleavage. Transglycosylation involves the breaking of the 1,4-β-glycosidic bond and the formation of a new bridging bond between C_1_ and the C_6_ hydroxyl group, yielding a chain end with levoglucosan (LG) [90]. The observed reaction step represents non-catalyzed transglycosylation at higher temperatures. Considering the obtained kinetic triplet for the studied reaction step (A→B) (Table 4), we may truly suppose that the production of LG (1,6-anhydro-β-D-glucopyranose) (as a reactive intermediate specie) takes place through the concerted one-step transglycosylation mechanism, which includes the simultaneous formation of the C_6_‒O‒C_1_ ether bridge and the breaking of the glycosidic bond. The described concerted mechanism has been calculated to have an activation barrier in the range between 192.464 kJ mol^−1^ and 232.212 kJ·mol^−1^ [90,91], which is lower than the barriers for a homolytic/heterolytic cleavage. Thus, the reaction A→B within the consecutive mechanism described by Equation (15), typifying the primary reaction in the cellulose conversion. This reaction is strongly favored by the operating conditions, such as the temperature of the heat source, the heat flux density, etc.

The following reaction, B→C, occurs in the much higher temperature region and represents the secondary reaction in the cellulose conversion. Considering the previous step (A→B), where we have a higher reaction order (n = 2.547 (Table 4)), the function f(α) = (1 − α)^n^ (Appendix A—Table A1) will decrease faster with conversion, α. This indicates with the transition of reactant that if the reaction has a higher reaction order, then the reaction rate will decrease faster (compared, for example, with the second-order reaction, n = 2). The higher the order of the reaction than 2, the more rapidly the reaction will decelerate with the transition of the reactant. In contrast to this case, the reaction B→C has an accelerating character, where the reaction rate increases with the generation of the product. This means that the conversion of LG (“B”) into the product (“C”) is characterized with the consumption of LG and the increase in product(s) yield. Consequently, the consumption of LG is characterized by the n-th order reaction (~4.764) throughout the autocatalysis (Cn model), and with activation energy (E) of 201.956 kJ mol^−1^ (Table 4). It should be emphasized that a degradation reaction with higher activation energy is possible to be autocatalytic, but in our specific case, the question arises about the classification of the autocatalytic strength.

For the established kinetic model, Cn, reaction B→C is governed actually by two paths, one that is an n-th order reaction, and another that is a autocatalytic reaction. It should be pointed out that in this case, based on the form of Cn model, B→C reaction is not catalyzed by one of its products, already by foreign factor, for example, by those present inorganics in the CAFB sample. Therefore, based on these facts, we aim to gain knowledge of the kinetic profiles of the products of the examined reaction. The catalytic behavior of the considered reaction is manifested through the Brønsted acidities of TiO_2_ (Brønsted acid catalyst) [92], which is present in the CAFB sample. Namely, the obtained kinetic model describes two intrinsic reaction pathways for the levoglucosan (LG) dehydration into the final products, characterized by the same E-value but with different “frequency” factors, which can be represented by Equation (21):
(21)
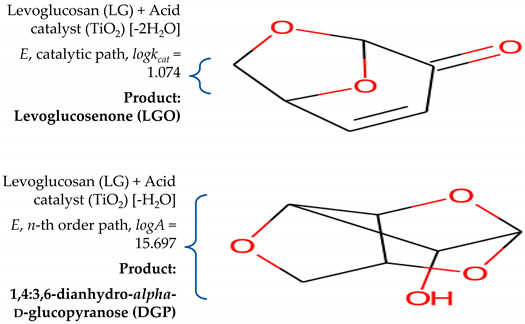



Therefore, reaction B→C, within the consecutive reaction steps (Equation (15)), represents the dehydration of LG which proceeds through two intrinsic steps, described by the Cn kinetic model with parameters listed in Table 4. The qualitative approach for determining the autocatalytic strength can represent an estimate of the ratio k_cat_/T* (where logk_cat_ = 1.074 ≝ k_cat_ = 11.858, and T* is the peak temperature at the maximum rate of B→C reaction at the different heating rates), and it is distributed between 0 and 1 [93]. Considering the T^*^ values at the various heating rates, β (5.1 K/min: 324 °C, 10.5 K/min: 335 °C, 21.5 K/min: 348 °C, and 32.8 K/min: 352 °C), the ratios k_cat_/T^*^ between 2 × 10^−2^ and 1.9 × 10^−2^ were obtained. Therefore, based on the estimated k_cat_/T* values, the less strong autocatalytic strength [93] (Equation (21)) was identified.

Based on the obtained results, we can clearly distinguish a sustainable strategy for the production of the attractive CAFB-derived platform molecule—levoglucosenone (LGO), which can be used for the renewable production of pharmaceuticals and commodity chemicals, as well as the production of promising anhydrosugar, such as 1,4:3,6-dianhydro-alpha-D-glucopyranose (DGP), which can be used as a versatile starting material not only in carbohydrate chemistry but also for the synthesis of non-carbohydrate and non-natural compounds [94].

Considering both reaction steps inside the sequential mechanism described by Equation (15), reaction A→B occurs at the conversion value of α ~ 25%, while reaction B→C takes place at a higher conversion of α ~ 71%, if the above results are compared with those obtained from the isoconversional kinetic analysis (Regions B and C) (Figure 6a,b). Finally, the contributions of reaction steps A→B and B→C (Equation (15)) to the entire combustion process of the CAFB sample are 31.4% and 39.9% (Table 4).

**Figure 10:**
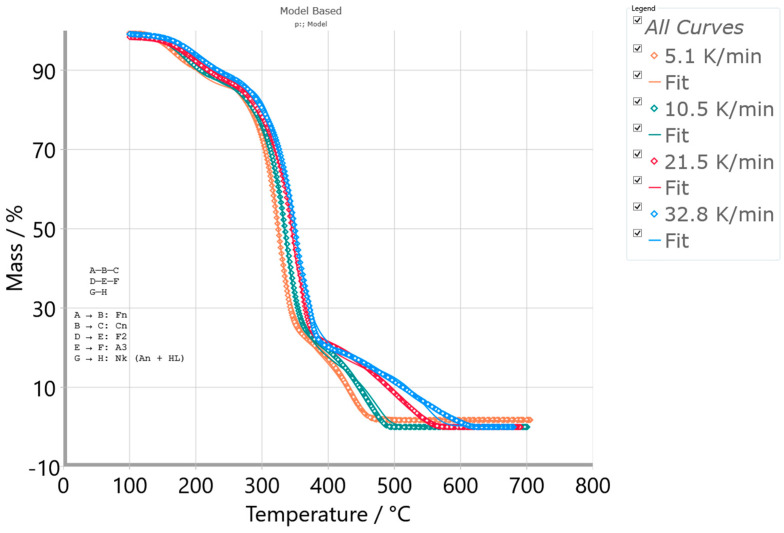
Model-based data fit to the experimental TG–signals at the different heating rates for the multi-step combustion process of CAFB sample (R^2^ = 0.99980).

Figure 10 shows the comparison of p:, Model with the experimental ones at various heating rates (5.1, 10.5, 21.5, and 32.8 K/min) for the combustion process of the CAFB sample.

It can be seen from Figure 10 that at all heating rates, the high value of R^2^ is obtained (R^2^ = 0.99980) where the model curves (fit) almost completely drown through the experimental points. Therefore, we can conclude that in order to obtain a reliable (decent) model-based prediction of the investigated process, the R^2^ value should be approximately 0.999, and in our case, this is unambiguously fulfilled.

**Figure 11:**
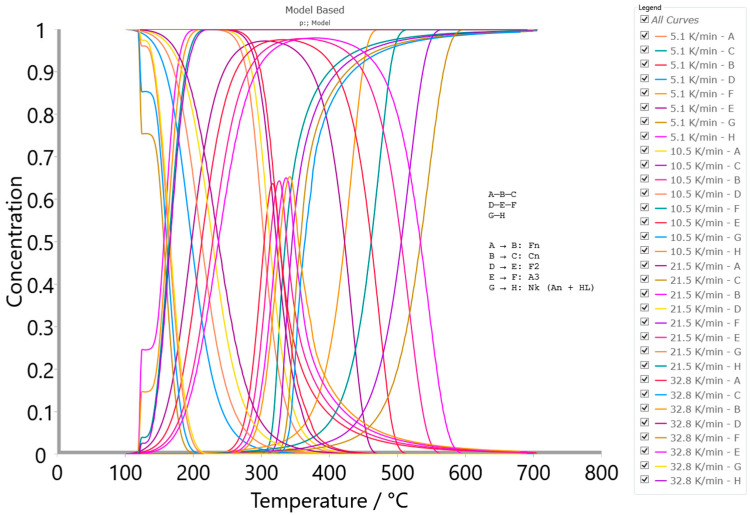
Evolution of concentration profiles of all chemical species that participate in the p:, Model (Equations (15)–(17)), which describes the complex non-isothermal combustion process of CAFB sample.

Figure 11 shows the relative change in the concentrations (normalized to “conversion” between 0 and 1) of all involved chemical species in proposed p:, Model, during the combustion process of CAFB sample.

The results presented in Figure 11 are in full agreement with the characteristics described for each elementary step within the proposed p:, Model scheme, highlighted under the items (a), (b), and (c) (see above).

According to the corresponding relationships between the values of activation energy (E) for elementary reaction steps, within the consecutive mechanisms described by Equations (15) and (16) (Table 4) and the applied heating rates, the rate-controlling steps can be determined. If we consider consecutive reaction steps $A\;\overset{k_{1}}{\to }\;B\;\overset{k_{2}}{\to }\;C$, at the low heating rate (5.1 K/min), it was found that k_1_ >> k_2_ (the rate constants are as follows: k_1_ = 0.041 s^−1^ and k_2_ = 0.009 s^−1^); therefore, elementary step B→C represents the rate-controlling step. This step was described by the kinetic model Cn, where the ratio of the rate constants of the n-th order and catalytic paths (k_n-th_/k_cat_) is very high (~×10^14^), indicating that the effective rate constant is high, and this suggests favoring the production of LGO with regards to DGP (Equation (21)). It should be noted a that similar situation holds for the high heating rate (32.8 K/min).

As for the consecutive reaction step $D\;\overset{k_{1}^{*}}{\to }\;E\;\overset{k_{2}^{*}}{\to }\;F$ at the low heating rate (5.1 K/min), the following situation was observed: k_1_^*^ > k_2_^*^ (the rate constants are as follows: k_1_^*^ = 0.005 s^−1^ and k_2_^*^ = 4.11 × 10*^−^*^6^ s^−1^). In this case, there is no “neglect” of any of the considered steps, and it takes place as described under item b). For the high heating rate (32.8 K/min), there is a different situation. In the actual case, it was valid that k_1_^*^ >>> k_2_^*^ (k_1_^*^ = 7.556 s^−1^ and k_2_^*^ = 0.001 s^−1^); therefore, the above sequence can be approximated as E→F, representing a shift in the rate*-*determining step with the variation in reaction affinity. Namely, this corresponds to the event that at high heating rates, the consecutive chemical reactions proceed as the single-step reaction, in which we have an accumulation of acrolein. We can see that the heating rate has a very strong influence on the regulation of the reactivity during the combustion of CAFB sample, which is related to the separation of products, such as LGO and acrolein. The observed influence of the heating rate on the chemical reactivity of certain species is naturally reflected in the phenomena observed during the analysis of the ignition-burnout characteristics and combustion performances of the CAFB sample (see Section 3.2.1).

### 3.4. Statistical Fit Quality Comparison between Model-Free and Model-Based Methods/Models

For the statistical analysis, in addition to the best coefficient of determination (R^2^) and the F-test, the sum of dev. squares (S^2^), the mean residual (MR), and Student’s coefficient with 95% confidence intervals are also used. Details about these statistical quantities can be found elsewhere [72]. Table 5 shows the comparative statistical analysis results from different model-free methods and the model-based method (Friedman (FR), Vyazovkin (VY), numerical (NM) and p:, Model) for the non-isothermal combustion process of the CAFB sample.

Considering the values of the obtained statistical parameters (Table 5), there is the following order of methods/models according to the quality of fitting the experimental results: NM > FR > VY > p:, Model. Namely, NM and FR models show a similar quality of fitting from their data to experimental ones, while VY and p:, Model show good comparative qualities in the overall fitting results but still less than the aforementioned models. In general, all the presented methods are quite acceptable for the kinetic analysis of the given process, and the obtained fitting results indicate that they are suitable for explaining all physicochemical phenomena that occur during the thermo-chemical conversion of CAFB.

### 3.5. Results of Simulation Tests—Isothermal Prediction Analysis

In order to evaluate the CAFB sample with regard to its long-term stability, isothermal predictions at different temperatures were calculated over a time period of 1 year. Starting at 0 °C, we calculated mass loss in 20 °C steps up to 200 °C, which gives a comprehensive insight into the thermal stability of CAFB, ranging from frozen storage to high-temperature stress conditions, and of course, taking into account all the applied methods/models.

Table 6 lists the conditions set for simulation tests for all the applied methods/models (FR, VY, NM, and p:, Model).

**Figure 12:**
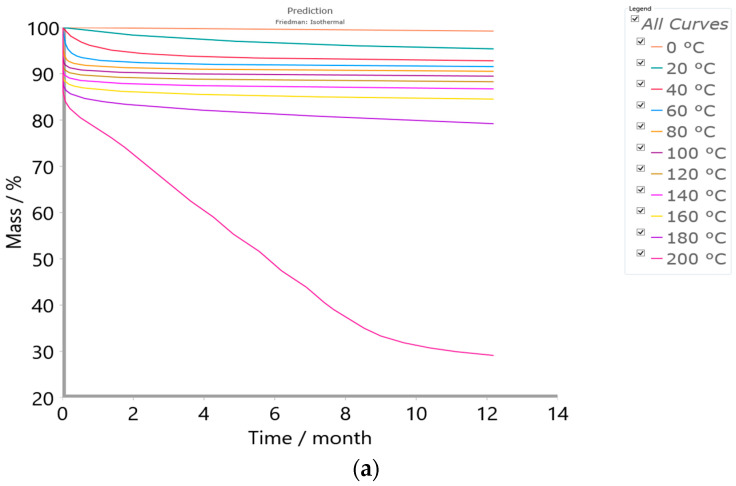

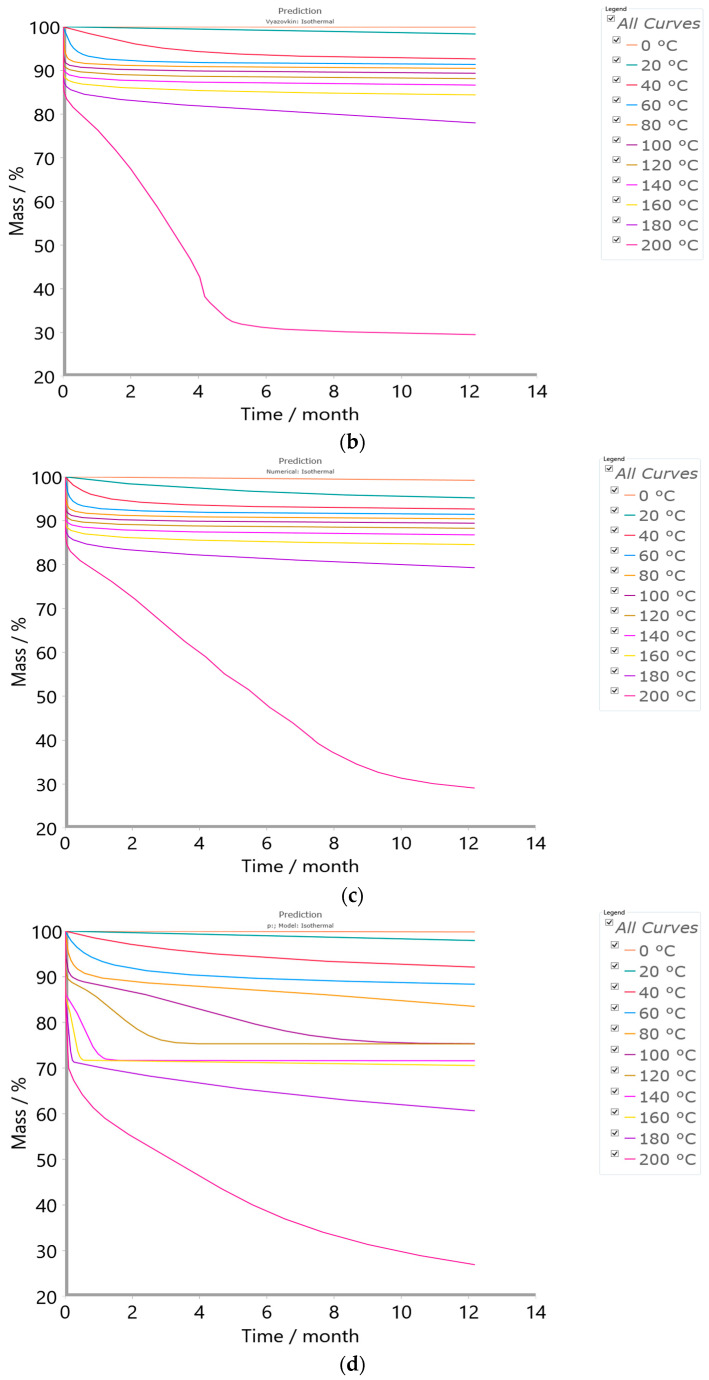
Isothermal predictions of the CAFB sample at different temperatures over a time period of 1 year, for the (**a**) Friedman (FR), (**b**) Vyazovkin (VY), (**c**) numerical (NM), and (**d**) p:, Model results.

The isothermal predictions for FR, VY, NM, and p:, Model results are depicted in Figure 12a–d, respectively.

Considering all the models, CAFB shows excellent thermal stability between 0 °C and 20 °C with no notable mass loss over the predicted time period. Even when stored at 60 °C, which has been measured as the maximum temperature for a sea shipping container, the predicted mass loss of CAFB is below 10%, which can be considered as fairly good stability of the sample (Figure 12). If the CAFB was exposed to higher temperature stress of 100 °C, the prediction shows a much gentler mass loss, which remains at approximately 10% total mass loss for isoconversional methods (Figure 12a–c), considering an entire 1 year. However, in the case of the model-based (p:, Model) method (Figure 12d), there is a steeper and more progressive mass loss, reaching a total mass loss of almost 24% after the time period of 1 year. Such differences arise due to divergence in the kinetic information provided by both kinetic approaches, where the p:, Model is more sensitive to the reactivity changes thermally induced in the considered material at the fixed temperatures. At the maximal temperature of 200 °C (highly demanding temperature conditions), there is a sudden drop in the mass loss, but the mass loss guideline traces are not the same among various isoconversional models, with that one related to the p:, Model (Figure 12). In the case of isoconversional models, the total mass loss when the CAFB is stored under 200 °C, amounts to approximately 73% over 1 year, while in the case of the p:, Model (model-based approach), the total mass loss amounts to approx. 75% (Figure 12). Therefore, it can be concluded that considering all the applied models, the CAFB sample shows fairly good thermal stability even when exposed to storage temperatures up to 60 °C, where the majority mass loss takes place during the first month. However, exposing the CAFB sample to higher temperatures, especially extremely high ones (200 °C), leads to the collapse of the thermal stability, causing its lowest long-term stability. This can be associated with a low value in the glass transition temperature (Table 4), as well as that the operating temperature of 200 °C is nearly the CAFB melting point (=240.695 °C (Table 4)). In view of the above discussion regarding the transformations that occur during the CAFB thermo-chemical conversion process, the isothermal simulations demonstrated the assessment of the thermal stability of CAFB sample, which can be acceptable in reality.

## 4. Conclusions

The presented kinetic study provides new insight into the reaction mechanism of the non-isothermal combustion process of cellulose acetate fibrous bundles (CAFB). The kinetic characteristics of the investigated system during its combustion were correlated to its general combustion properties and vital parameters attached to conversion performances. It was found that there is great influence from the process operational parameters, such as the temperature, i.e., the heating rate and the sample particle size, on the comprehensive combustion activities. It was shown that these properties were strongly related to the chemical content of the sample, which consists of major components such as cellulose diacetate (CDA), triacetin (TA-plasticizer), and titanium dioxide (TiO_2_) (delustering agent). It was established that slow heating leads to inadequate combustion (there is a residual ash that was identified), but the increase in the heating rate resulted in the positive response of the system to the ignition behavior. In addition, the CAFB showed superior burnout performance at the highest heating rate (32.8 K/min), where the presence of metals may improve the CAFB burnout performance. However, it was detected that the best combustion stability of the CAFB sample is achieved at the lowest heating rate (5.1 K/min). Therefore, as a concluding word, it was stated that the best comprehensive combustion characteristics of the CAFB sample are achieved at the lowest heating rate. A detailed kinetic analysis implemented through model-free (isoconversional) and model-based methods revealed that CAFB combustion proceeds as a multi-step process through several elementary reaction steps, which characterize the corresponding physicochemical changes described here for the first time. The proposed mechanistic scheme realistically described the changes within the system, from the morphological regulation and supramolecular architecture of the cellulose polymer until the production of energy carriers and platform chemicals, as well as the versatile starting materials for organic synthesis, when cellulose represents the “initial” reaction feedstock. The heating rate was found to be a key process parameter that governs the yield and separation of two essential but ecologically diverting compounds, such as levoglucosenone (LGO) (which is used for the renewable production of pharmaceuticals and commodity chemicals) and acrolein (which, in the observed conditions, is a toxin), from CAFB thermo-chemical conversion. Additionally, the performed isothermal predictions have shown that CAFB exhibits very good long-term stability at the temperature of 60 °C, corresponding to the storage in a sea shipping container. On the other hand, at high-temperature stress conditions (~200 °C), the CAFB manifests the collapse of thermal stability, causing its lowest long-term stability. The identified behavior of the CAFB sample in terms of thermal stability was related to its physical properties, such as the glass transition temperature (*T_g_*) and the melting temperature (*T_m_*) value positions.

## Figures and Tables

**Table 1:** Experimentally determined values of the cellulose diacetate (CDA) signal, then its fragments and corresponding theoretical values.

Chemical Formula	IUPAC Nomenclature	Experimental *m*/*z*	Theoretical *m*/*z*
[C_6_H_11_O_5_]^+^	(3S,4R,5S,6R)-3,4,5-trihydroxy-6-(hydroxymethyl)tetrahydro-2H-pyran-2-ylium	163.39	163.15
[C_6_H_11_O_6_]^+^	(3R,4S,5S,6R)-2,3,4,5-tetrahydroxy-6-(hydroxymethyl)tetrahydro-2H-pyran-2-ylium	173.39	179.15
[C_7_H_11_O_7_]^+^	(3R,4S,5S,6S)-3-acetoxy-2,4,5,6-tetrahydroxytetrahydro-2H-pyran-2-ylium	207.36	207.16
[C_20_H_29_O_15_]^+^	(3R,4S,5S,6R)-3-acetoxy-5-(((2S,3R,4S,5S,6R)-3-acetoxy-6-(acetoxymethyl)-4,5-dihydroxytetrahydro-2H-pyran-2-yl)oxy)-6-(acetoxymethyl)-2,4-dihydroxytetrahydro-2H-pyran-2-ylium	509.47	509.44

**Table 2:** Combustion characteristic temperatures and quantitative comprehensive combustion indices for CAFB sample.

*β* (K/min)	*T_i_* (°C)	*T_p_* (°C)	*T_b_* (°C)	−*R_p_* (%·min^−1^)	*D_i_* (%·min^−3^)	*D_b_* (%·min^−4^)	*C* (%·°C^−2^·min^−1^)	*S* (10^−9^·%^2^·°C^−3^·min^−2^)	*H_f_* (10^3^·°C)
5.1	295.59	325.24	470.70	1.221	4.124 × 10^−4^	2.844 × 10^−5^	1.397 × 10^−5^	4.167	1.174
10.5	307.80	336.67	486.03	1.135	1.341 × 10^−3^	1.885 × 10^−4^	1.197 × 10^−5^	3.455	1.251
21.5	321.35	350.14	563.64	1.047	4.006 × 10^−3^	9.606 × 10^−4^	1.013 × 10^−5^	2.256	1.378
32.8	326.09	354.11	611.50	1.007	7.488 × 10^−3^	2.513 × 10^−3^	9.466 × 10^−6^	1.846	1.441

**Table 3:** Mathematical description through rate-law differential equations of all elementary steps included in p:, Model.

Elementary Step	Model Annotation	Concentration Equation	Rate Equation	Kinetic Parameters and Exponents ^d^
*A*→*B*	F*n*	d*a*/d*t* = −d(*a−>b*)/dt d*b*/dt = d(*a−>b*)/dt − d(*b−>c*)/dt d*c*/dt = d(*b−>c*)/dt d*d*/dt = −d(*d−>e*)/dt d*e*/dt = d(*d−>e*)/dt − d(*e−>f*)/dt d*f*/dt = d(*e−>f*)/dt d*g*/dt = −d(*g−>h*)/dt d*h*/dt = d(*g−>h*)/dt	d(*a−>b*)/dt = *A*·*a^n^*·*exp*[−*E*/(*RT*)]	*A*,*E*,*n*(*n* ≡ reaction order)
*B*→*C*	C*n*		d(*b−>c*)/dt = *A*·*b^n^*·(1 + AutocatPreExp·*c*)·*exp*[−*E*/(*RT*)]	*A*,*E*,*n*(*n* ≡ reaction order), AutocatPreExp. ^e^
*D→E*	F2		d(*d−>e*)/dt = *A*·*d*^2^·*exp*[−*E*/(*RT*)]	*A*,*E*,*n*(reaction order, *n* = 2)
*E→F*	A3		d(*e−>f*)/d*t* = *A*·3*e*·[−ln(*e*)]^(2/3)^·*exp*[−*E*/(*RT*)]	*A*,*E*,*n*(*n* ≡ Avrami exponent)
*G*→*H*	Nk		d(*g−>*h)/dt = *A*·*n*·*g*·[−ln(*g*)]^[(*n* − 1)/*n*]^·*exp*[−U*/(*R*(*T*−(*T_g_* − 30)))]·*exp*[−*K*_G_·(*T* + *T_m_*)/(2·*T*^2^·(*T_m_* − *T*))] ^a, b, c^	*A*,*n* (*n* ≡ Avrami exponent), U*, *K*_G_

^a^ U* is the activation energy of the segmental jump (the activation energy necessary for the macromolecules to diffuse to the crystal phase in a melting state) (this parameter has the universal value of 6.300 kJ mol^−1^), while K_G_ is the nucleation parameter (in a function of the surface free energy; represents an activation energy of the nucleation for a crystal with the critical size), which relates to the fold and lateral surface energies (refer to [12]). ^b^ T_g_—the glass transition temperature (°C). ^c^ T_m_—the melting temperature (°C). ^d^ A—the pre-exponential factor of elementary reaction step (s^−1^), and E—the activation energy of elementary reaction step (kJ·mol^−1^). ^e^ AutocatPreExp. represents the weight factor (autocatalysis factor), k_cat_ (Table A1), or it can be said that it is a frequency factor for catalytic reaction path.

**Table 4:** The full reaction mechanism scheme—p:, Model, for the CAFB non-isothermal combustion process.

Model Scheme: *A*─*B*─*C* *D*─*E*─*F* *G*─*H*	
**Step: *A*→*B* (F*n*); Δ*T* = 260 °C − 450 °C**	
Activation Energy (*E*) (kJ·mol^−1^)	229.505
Log(PreExp) (*logA*), *A* (s^−1^)	18.760
React. Order, *n*	2.547
Contribution	0.314 (31.4%)
**Step: *B*→*C* (C*n*); Δ*T* = 300 °C − 700 °C**	
Activation Energy (*E*) (kJ·mol^−1^)	201.956
Log(PreExp) (*logA*), *A* (s^−1^)	15.697
React. Order, *n*	4.764
Log(AutocatPreExp), *k_cat_*	1.074
Contribution	0.399 (39.9%)
**Step: *D*→*E* (F2); Δ*T* = 100 °C − 350 °C**	
Activation Energy (*E*) (kJ·mol^−1^)	86.526
Log(PreExp) (*logA*), *A* (s^−1^)	7.288
Contribution	0.110 (11%)
**Step: *E*→*F* (A3); Δ*T* = 350 °C − 700 °C**	
Activation Energy (*E*) (kJ·mol^−1^)	66.944
Log(PreExp) (*logA*), *A* (s^−1^)	2.068
Contribution	0.140 (14%)
**Step: *G*→*H* (Nakamura, Nk); Δ*T* = 120 °C − 260 °C ^c^**	
Nakamura, *K*_G_ (×1000)) (K^2^)	−30.219
Log(PreExp) (*logA*), *A* (s^−1^)	2.411
Dimension (*n*)	0.374
Temp. Melting, *T_m_* (°C) ^a^	240.695
Temp. Glass, *T_g_* (°C) ^b^	120.000
U* (kJ·mol^−1^)	6.300
Contribution	0.037 (3.7%)

^a^ Calculated T_m_, which was confirmed from the Nakamura (Nk) best optimized parameters. ^b^ Calculated T_g_, which was confirmed from the Nakamura (Nk) best optimized parameters. ^c^ p:, Model considers the overlapping effect among elementary steps during CAFB conversion.

**Table 5:** Comparative statistical analysis results (a statistical fit quality) of the different model-free (isoconversional) methods (FR, VY, and NM) and the model-based method (p:, Model), for non-isothermal combustion of CAFB.

Method/Model	Fit to	R^2^	Sum of Dev. Squares (S^2^)	Mean Residual (MR)	Students Coef. 95%	F-Test
Friedman (FR)	TG-signal	0.99990	612.679	0.359	1.961	1.145
Vyazovkin (VY)	TG-signal	0.99987	754.574	0.367	1.961	1.410
Numerical (NM)	TG-signal	0.99991	535.014	0.354	1.961	1.000
p:, Model	TG-signal	0.99980	836.609	0.489	1.961	1.501

**Table 6:** Conditions given for implementation of isothermal predictions, including FR, VY, NM, and p:, Model results.

Method/Model FR/VY/NM/p:, Model	
Minimal temperature (°C)	0
Maximal temperature (°C)	200
Temperature step (°C)	20
Time (year)	1

## Data Availability

Data are contained within the article.

## Appendix A

**Table A1:** Kinetic model functions (in differential form of the analytical kinetic functions, *f*(*α*)) used in the current work for computational procedure in the model-based analysis.

Model	Symbol	*f*(α)
Phase boundary-controlled reaction (contracting disk, 1D)	R1/F0	(1 − *α*)^0^
Phase boundary-controlled reaction (contracting area, 2D)	R2	2·(1 − *α*)^1/2^
Phase boundary-controlled reaction (contracting volume, 3D)	R3	3·(1 − *α*)^2/3^
Random nucleation, unimolecular decay law, first order chemical reaction	F1	(1 − *α*)
Second order chemical reaction	F2	(1 − *α*)^2^
*n*-th order chemical reaction (*n* ≠ 1)	F*n*	(1 − *α*)*^n^*
Two-dimensional growth of nuclei (Avrami equation)	A2	2·(1 − *α*)[−ln(1 − *α*)]^1/2^
Three-dimensional growth of nuclei (Avrami equation)	A3	3·(1 − *α*)[−ln(1 − *α*)]^2/3^
*n*-dimensional nucleation (Avrami–Erofeev equation)	A*n*	*n*·(1 − *α*)[−ln(1 − *α*)]^1−1/*n*^
One-dimensional diffusion, parabola law	D1	1/2*α*
Two-dimensional diffusion, Valensi equation	D2	1/[−ln(1 − *α*)]
Three-dimensional diffusion, Jander equation	D3	(3/2)(1 − *α*)^2/3^/[1 − (1 − *α*)^1/3^]
Three-dimensional diffusion, Ginstling–Brounstein	D4	(3/2)/[(1 − *α*)^−1/3^ − 1]
Prout–Tompkins equation	B1	(1 − *α*)·*α*
Expanded Prout–Tompkins equation	B*na*	(1 − *α*)*^n^·α^a^*
First order with autocatalysis	C1	(1 + *k_cat_*·*α*)(1 − *α*)
*n*-th order with autocatalysis	C*n*	(1 + *k_cat_*·*α*)(1 − *α*)*^n^*
*n*-th order and *m*-power with autocatalysis	C*nm*	(1 − *α*)*^n^*·*α^m^*
Expanded Šestak–Berggren (SB) equation	SB*nmq*	(1 − *α*)*^n^*·*α^m^*·[−ln(1 – *α*)]*^q^*
Kamal–Sourour equation	KS	(*k*_1_ + *k*_2_·*α^m^*)(1 − *α*)*^n^*
Nakamura crystallization	Nk (A*n* + H-L)	*f*(*α*)·*K*(*T*), *f*(*α*) = *n*·(1 − *α*)[−ln(1 − *α*)]^1−1/*n*^, where for analytical dependence of the rate constant *K*(*T*), Hoffman−Lauritzen (H−L) theory is used (non−Arrhenius).
Šestak–Berggren crystallization or Sbirrazzuoli crystallization	(SBC/SC) (SB + H-L)	*f*(*α*)·*K*(*T*), *f*(*α*) = (1 − *α*)*^n^*·*α^m^*·[−ln(1 – *α*)]*^q^*, where for analytical dependence of the rate constant *K*(*T*), Hoffman−Lauritzen (H−L) theory is used (non−Arrhenius)
